# Comparison between Fluorescence Imaging and Elemental Analysis to Determine Biodistribution of Inorganic Nanoparticles with Strong Light Absorption

**DOI:** 10.1021/acsami.1c11875

**Published:** 2021-08-16

**Authors:** Konstantin Tamarov, Julie Tzu-Wen Wang, Juuso Kari, Emilia Happonen, Ilkka Vesavaara, Matti Niemelä, Paavo Perämäki, Khuloud T. Al-Jamal, Wujun Xu, Vesa-Pekka Lehto

**Affiliations:** † Department of Applied Physics, Faculty of Science and Forestry, University of Eastern Finland, Kuopio 70211, Finland; ‡ School of Cancer and Pharmaceutical Sciences, Faculty of Life Sciences & Medicine, King’s College London, London SE1 9NH, U.K.; § Research Unit of Sustainable Chemistry, University of Oulu, Oulu 90570, Finland

**Keywords:** black porous silicon, nanoparticles, size effect, biodistribution, PEGylation, surface modification

## Abstract

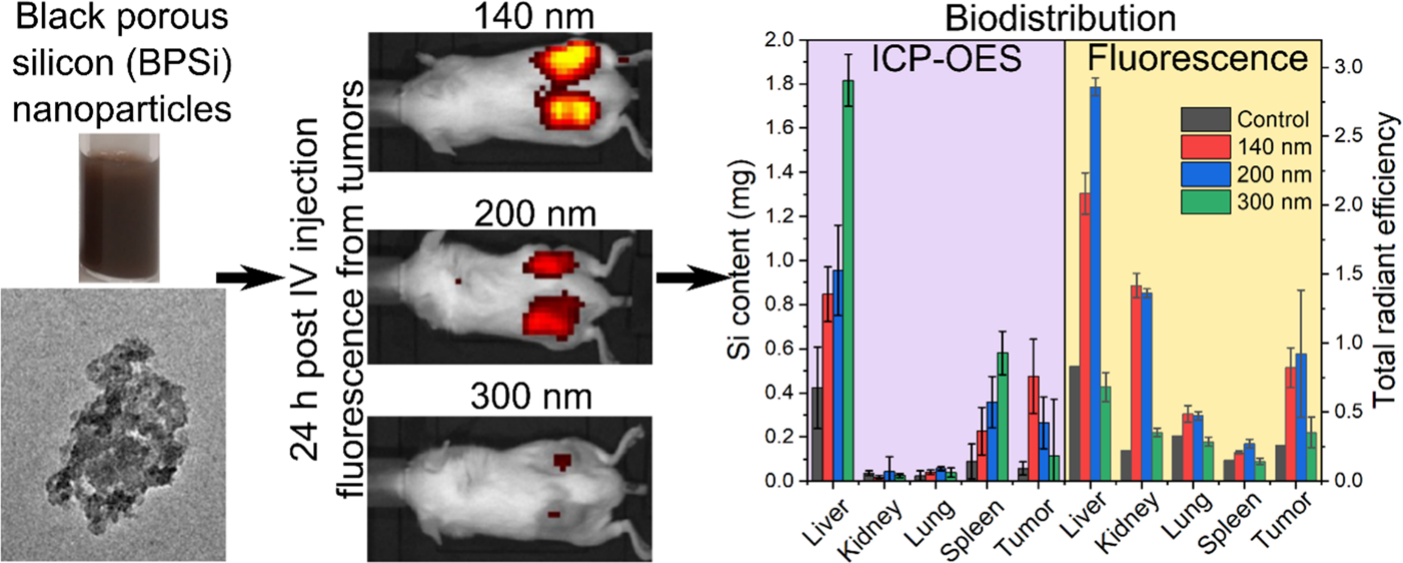

Black porous silicon nanoparticles (BPSi NPs) are known as highly efficient infrared light absorbers that are well-suitable for photothermal therapy (PTT) and photoacoustic imaging (PAI). PTT and PAI require a sufficient number of effectively light-absorbing NPs to be accumulated in tumor after intravenous administration. Herein, biodistribution of PEGylated BPSi NPs with different sizes (i.e., 140, 200, and 300 nm in diameter) is investigated after intravenous administration in mice. BPSi NPs were conjugated with fluorescent dyes Cy5.5 and Cy7.5 to track them *in vitro* and *in vivo*, respectively. Optical imaging with an *in vivo* imaging system (IVIS) was found to be an inadequate technique to assess the biodistribution of the dye-labeled BPSi NPs *in vivo* because the intrinsic strong absorbance of the BPSi NPs interfered fluorescence detection. This challenge was resolved via the use of inductively coupled plasma optical emission spectrometry to analyze *ex vivo* the silicon content in different tissues and tumors. The results indicated that most of the polyethylene glycol-coated BPSi NPs were found to accumulate in the liver and spleen after intravenous injection. The smallest 140 nm particles accumulated the most in tumors at an amount of 9.5 ± 3.4% of the injected dose (concentration of 0.18 ± 0.08 mg/mL), the amount known to produce sufficient heat for cancer PTT. Furthermore, the findings from the present study also suggest that techniques other than optical imaging should be considered to study the organ biodistribution of NPs with strong light absorbance properties.

## Introduction

An efficient and safe cancer treatment with nanomedicines should ideally exhibit preferential tumor accumulation, thus lowering the administration dose and reducing side effects in healthy organs at the same time.^1^ Effective accumulation in tumors is particularly important for the treatment modalities that rely on external triggers, such as magnetic hyperthermia^2^ and hyperthermia induced by radiofrequency radiation,^3−5^ ultrasound,^6,7^ or light [photothermal therapy (PTT)].^8^ The latter one especially relies on the effective accumulation because the penetration depth of light is rather limited even in the most transparent near-infrared window in biological tissues.

There are two main approaches to direct nanoparticles (NPs) to tumors. The first one, active targeting, relies on the surface modifications of NPs to target specific receptors expressed by tumor cells. Strategies include modification of the NP surface with targeting antibody, aptamer, and ligand molecules to achieve specific accumulation in tumors.^9^ The exposed targeting moieties, however, can be hidden under a layer of protein coronas due to opsonization of NPs.^10^ The opsonization may render the targeting ability of the moieties and additionally increase the recognition of NPs by the immune system with subsequent clearance. Furthermore, targeting moieties themselves can be recognized as foreign bodies and cleared from the blood. Recently, active targeting has been questioned^11^ and it has been demonstrated that active targeting shows insignificant benefit compared to passive targeting.^12^

The second approach, passive targeting, is based on the enhanced permeability and retention (EPR)^13^ effect of increased vascular permeability for tumors that develop leaky blood vessel endothelium. To achieve high tumor accumulation by the EPR effect, NPs have to circulate in blood as long as possible and to be small enough for extravasation into tumor. The long systemic presence is most commonly realized with polyethylene glycol (PEG) coatings,^10^ which reduces opsonization and prolongs blood circulation time for hours compared to uncoated NPs.^14^ Size dependence of tumor accumulation was observed for PEG-coated spherical gold,^15,16^ polymeric,^17,18^ and silica NPs.^19,20^ The general trend for all types of the NPs is that with the decrease in size, the tumor accumulation is increased. Additionally, shape was proposed to influence the tumor availability of porous silicon microparticles.^21^

Black porous silicon (BPSi) is a new type of photothermal conversion agent with strong light absorption. Compared with other inorganic counterparts, such as Au, graphene, and CuS, the BPSi NPs have unique features of high surface area, large pore volume, and excellent biocompatibility.^22,23^ The BPSi NPs have been applied in PTT^24^ and photoacoustic tomography.^25^ We have previously demonstrated the feasibility of BPSi NPs for cancer PTT following intratumoral injection.^24^ However, the biodistribution of the BPSi NPs after intravenous injection was still unrevealed. In the present study, we investigate their size-dependent biological behavior *in vitro* and *in vivo*. The effects of the different particle sizes (i.e., 140, 200, and 300 nm in diameter) and surface modifications of BPSi NPs on their cellular uptake and biocompatibility were investigated *in vitro*. We further studied their organ biodistribution and passive tumor targeting (syngeneic CT26 tumor model) *in vivo* qualitatively with optical imaging and quantitatively with inductively coupled plasma-optical emission mass spectrometry (ICP-OES MS) analysis after intravenous injection.

## Experimental Section

### Preparation of the NPs and Surface Modifications

The BPSi NPs were prepared with the method developed before.^24^ After the preparation, the surface of BPSi NPs is hydrogen-terminated. To make it hydrophilic and enable further modification, the surface of NPs was oxidized by immersing 20 mg of them into 30 mL of HCl/H_2_O_2_/H_2_O (volume ratio: 1/1/5) solution to produce surface −OH groups. The solution was stirred and kept at 90 °C for 30 min, after which the particles were rinsed with water and ethanol by repeating high speed centrifugation and sonication–redispersion steps three times before final storage in ethanol. The obtained oxidized NPs were noted as BPSi-OH. Next, BPSi-OH NPs were functionalized with amine groups with (3-aminopropyl) triethoxysilane (APTES, Acros Organics BVBA, China, 99%) to which cyanine dye (Cy5.5-NHS or Cy7.5-NHS, Lumiprobe GmbH, Germany) was conjugated via the reaction between NHS and the amine group. To do that, 0.25 mg of either Cy5.5-NHS or Cy7.5-NHS was first dispersed in 0.5 mL of ethanol by sonication. Then, 13 mg of BPSi-OH NPs dispersed in 1 mL of ethanol was placed into a 20 mL glass vial, followed by pouring the dye solution. Finally, 10 μL of APTES was added and the mixture was stirred for 40 min at 65 °C. The vial was kept closed to prevent ethanol evaporation. The resulting BPSi-Cy5.5/Cy7.5 NPs were washed with ethanol three times. The residual amine groups were capped by the reaction of BPSi-Cy5.5/Cy7.5 NPs with 20 mg of succinic anhydride (referred as COOH-BPSi-Cy5.5/Cy7.5) in 2 mL of ethanol for 16 h at room temperature. Finally, the NPs were functionalized with two different PEG molecules (PEG 2.0 kDa and PEG 0.5 kDa, Hunan Huateng Pharmaceutical Co. Ltd., China and Gelest Inc., USA, respectively) as described before^26^ and referred to as PEG-BPSi-Cy5.5/Cy7.5. Briefly, for 13 mg of BPSi-Cy5.5/Cy7.5 NPs, 130 mg of PEG 2.0 kDa was first dissolved in 1.5 mg of anhydrous toluene by sonication and subsequently poured into an Erlenmeyer flask. Under stirring, 260 μL of PEG 0.5 kDa was then added to the flask, followed by the NP dispersion in 2 mL of ethanol. Afterwards, the flask was heated to 65 °C with a N_2_ flow for 20 min to evaporate ethanol, and subsequently, additional 2 mL of toluene was added into the flask, which was then sealed and left for 18 h for PEG molecules to react with −OH groups on the surface of BPSi-Cy5.5/Cy7.5 NPs. Finally, toluene was evaporated with N_2_ for 20 min and PEG-BPSi-Cy5.5/Cy7.5 was washed three times with ethanol to remove unreacted PEGs and stored in the fridge.

### Size Separation

To study the effect of different diameters, the NPs were centrifuged at different speeds after surface modifications to get a mean diameter of 140, 200, and 300 nm. First, the mixture of all sizes was centrifuged in ethanol for 20 min at 3000 *g* in water to separate the 140 nm NPs that stayed in the supernatant. Afterward, the sediment was resuspended in ethanol by sonication and centrifuged for 15 min at 1200 *g* to separate 200 nm NPs. Finally, large particles were precipitated by 2 min centrifugation at 300 *g*. The NPs that stayed in the suspension were then around 300 nm. Each centrifugation was repeated 2–3 times to ensure that most of the NPs of a certain size were collected.

### Characterization

#### Transmission Electron Microscopy

Transmission electron microscopy (TEM) images were acquired with JEOL JEM-2100F (JEOL Ltd., Japan) after drying a 2.5 μL drop of PEG-BPSi-Cy7.5 dispersed in ethanol on a 400 mesh carbon-coated copper grid (Agar Scientific Ltd., UK).

#### UV–Vis–NIR Absorption

Light absorption measurements in the range of 400–900 nm were performed using a Jasko V-530 (Jasko, Japan) instrument at a high scanning rate. One milliliter of BPSi-OH water suspension (0.05 mg/mL) was placed into a quartz cuvette; water was used as the background.

#### Surface Modifications

Surface modifications were studied with Fourier transform infrared spectroscopy (FTIR, Thermo Nicolet iS50, Thermo Fisher Scientific Inc., USA) and thermogravimetric analysis (TGA, NETZSCH TG 209F1 Libra, Netzsch Holding, Germany). TGA was performed under 20 mL/min N_2_ flow to avoid oxidation using the following sequence: (1) ramp 20 K/min to 80 °C; (2) keep 20 min at 80 °C to evaporate water; and (3) ramp 20 K/min to 900 °C. The surface modifications of organic compounds such as APTES and PEG were decomposed and evaporated during heating in a N_2_ atmosphere, while the inorganic porous silicon was kept. Thus, the mass losses for the NPs from different stages of surface modifications were compared to determine the amount of conjugated APTES, dye, PEGs, and −COOH groups.

#### Colloidal Stability

For colloidal stability studies, 0.1 mg/mL suspensions were incubated at 37 °C in deionized water, 5% mannitol solution (d-mannitol, Sigma-Aldrich, France), and 1:1 phosphate-buffered saline (PBS)/plasma solution (volume ratio). At selected time points, 0.1 mL of particle suspension was diluted with 0.9 mL of water to measure size distributions and ζ potential using a ZetaSizer Nano ZS (Malvern Panalytical Ltd., UK) instrument. The same instrument was used to determine particle sizes right after size separations by centrifuging. The number of particles for each of the three particle sizes was analyzed using NP tracking analysis (NanoSight LM10, Malvern Panalytical Ltd., UK). Water suspensions of PEG-BPSi-Cy7.5 at 0.2 μg/mL were measured three times, and the average number of particles was calculated.

The appearance of aggregates for the NPs without PEG coating was additionally visualized by TEM imaging performed using JEOL JEM-2100F. Herein, NPs were dispersed in 50 mM pH 7.0 PBS buffer (VWR chemicals) at a concentration of 0.5 mg/mL and subsequently incubated in 37 °C for 4 h. 4 h time point was selected to observe the aggregation difference between BPSi-NH_2_-Cy5.5 and COOH-BPSi-Cy5.5. After 4 h of incubation, 100 μL of suspension was slowly dropped on 400 mesh holy carbon copper TEM grid (Agar Scientific Ltd., UK) located on a paper tissue which consumed all the excess liquid. The grid was then kept for 5 min to dry.

### *In Vitro* Evaluation

#### Cell Internalization

Internalization experiments were studied with CT26 mouse colon cancer cells and RAW 264.7 mouse macrophage cells. Twenty thousand CT26 or RAW 264.7 cells were cultured on 8-well plates (Ibidi μ-Slide 8 Well for confocal microscopy) in 0.2 ml of RPMI 1640 or DMEM (Biowest SAS, France) medium, respectively. After the 24 h incubation for cell attachment, the cells were washed with 0.2 mL of PBS buffer solution and with 0.2 mL of cell medium. Then, BPSi-NH2-Cy5.5, COOH-BPSi-Cy5.5, and PEG-BPSi-Cy5.5, dispersed in 0.2 mL of cell culture medium, were added into each well at 0.05 mg/mL final concentration. Cells were washed twice with buffer solution (Hank’s Balanced Salt Solution, Biowest SAS, France) after the incubation at 37 °C for 24 h. Then, cell membranes were stained with CellMask Green (Thermo Fisher Scientific Inc., USA) at a concentration of 1 μg/mL for 8 min. After that, they were washed with buffer solution and cell medium to remove free dye. The internalization of the NPs was inspected with a Zeiss LSM 700 (Zeiss Group, Germany) laser scanning confocal microscope with two channels for Cy 5.5 and CellMask Green.

The aggregation and internalization of 140 nm NPs into CT26 cells was further examined using TEM imaging. A total of 10,000 CT26 cells per well were attached to the bottom of a 24-well plate by 24 h of incubation. Then, BPSi-NH_2_-Cy5.5, COOH-BPSi-Cy5.5, and PEG-BPSi-Cy5.5 at a concentration of 0.05 mg/mL were added to cells and incubated for 24 h. Finally, the noninternalized NPs were removed from cells by washing once with cell medium and fixed in epoxy resin. The fixation was performed according to the following protocol. Cells in the well plate were first fixed with 2% glutaraldehyde in 0.1 M phosphate buffer (pH 7.4) for 1 h at RT, followed by 2 × 5 min wash in 0.1 M phosphate buffer (pH 7.4). Cells were then postfixed with 1% osmiumtetraoxide in 0.1 M phosphate buffer (pH 7.4) for 1 h at RT, followed by 2 × 5 min wash in 0.1 M phosphate buffer (pH 7.4). Next, cells were dehydrated in 70% ethanol for 5 min, followed by 90% ethanol for 5 min, and then in 94% ethanol and absolute ethanol for 2 × 5 min. After that, epoxy resin infiltration was performed for 2 h in the 1:1 mixture of Epon and absolute ethanol with subsequent removal of the mixture. Finally, Beem capsules filled with Epon were placed on top of the cells; they were incubated for 2 h at RT for further infiltration of Epon, followed by 24 h polymerization at 60 °C. The polymerized block was snapped out of the well by bending and sliced to 60 nm ultrathin sections at different depths. The slices were then placed on TEM grid for imaging.

#### Viability

Cell viability experiments were used to study cytotoxicity of CT26 cancer cells exposed to BPSi NPs with different particle sizes and surface coatings. To enhance the cell attachment on the 96-well plate, the surface of the plate was coated with poly-l-lysine solution (0.01%). Poly-l-lysine solution (25 μL) was added to the bottom of the wells and let to dry in an incubator (37 °C) for 2 h. The wells were washed one time with 0.1 mL of PBS buffer solution, and then, cells (5000 cells/well) were added for cell culture. The cells were grown for 48 h to attach the cells on the bottom of the wells. The NPs with different surface coatings (BPSi-NH2-Cy5.5, COOH-BPSi-Cy5.5, and PEG-BPSi-Cy5.5) and sizes (140, 200, and 300 nm) were washed with deionized H_2_O and then dispersed in cell culture medium at four different concentrations (0.05, 0.1, 0.2, and 0.5 mg/mL). A total of 0.1 mL of each sample was added into each well (*n* = 4) and they were incubated for 24 h. Then, the cells were washed once with the medium and incubated with fresh medium for another 24 h. Cells incubated in fresh medium or treated with 0.1 mL of 1% of Triton X-100 were used as positive and negative controls, respectively, for the same period of time. At the end, the cells were washed with buffer solution and cell culture medium. The cell viability was measured with CellTiter-Glo assay (Promega Inc., USA) according to the manufacturer’s instructions using a Victor3 (PerkinElmer Inc., USA) device.

### *In Vivo* Studies in CT26 Tumor-Bearing Mice

*In vivo* organ biodistribution and biocompatibility studies were performed under the authority of project and personal licenses granted by the UK Home Office and the UKCCCR Guidelines (1998). Tumors were established by subcutaneously injecting CT26 cells (1 × 10^6^ cells) at the lower flanks (left and right) of female BALB/c mice (2 tumors per animal). After 12 days of tumor growth, animals were divided into five groups (three mice per group): control, free Cy7.5 dye, and PEG-BPSi-Cy7.5 of three different sizes (140, 200, and 300 nm in diameter). For the PEG-BPSi-Cy7.5 groups, mice received i.v. injection of the NPs (5 mg/mouse) suspended in 5% mannitol solution. For the control and free dye groups, mice were injected with 5% mannitol solution or Cy7.5 (0.015 mg/mouse) suspended in 5% mannitol solution, respectively. 5% mannitol solution is approximately isotonic and suitable for intravenous injection alone or with other medications. Furthermore, this solution does not induce fast aggregation of NPs compared to, for example, saline or PBS. Fluorescence imaging was performed at 1, 4, and 24 h postinjection using an IVIS Lumina III *in vivo* imaging system (PerkinElmer Inc., USA). After 24 h, animals were sacrificed and selected vital organs (brain, heart, lung, liver, spleen, kidney, stomach, intestine, and tumors) were excised for *ex vivo* imaging. The analysis of PEG-BPSi-Cy7.5 NPs *ex vivo* was then performed using IVIS software. The appropriate regions of interest (ROIs) that surround the organs were drawn using the hand, after which the software calculated the total radiant efficiency for each ROI. Thus, the total radiant efficiency is proportional both to the surface area of the organ in the image (area of ROI) and to the fluorescence signal from each pixel in the ROI. Finally, parts of the organs and tumors were fixed in 10% neutral buffered formalin for histological assessment. After fixing, samples were wax-embedded and sectioned for hematoxylin and eosin (H&E) or neutral red staining according to standard histological protocols at the Royal Veterinary College, UK. All stained sections were analyzed using a Leica DM 1000 LED Microscope (Leica Microsystems, UK) coupled with a CDD digital camera (Qimaging, UK).

### ICP-OES Analysis of Si Content

Si content in major organs (liver, spleen, kidney, lung, and tumor) was quantified by ICP-OES. First, empty microwave vessels were acid-washed in the microwave oven with 5 mL of HF and 3 mL of HNO_3_ to remove possible Si contaminations. Then, organs were transferred to the vessels and sample masses were recorded. A total of 5 mL of nitric acid (Fisher, TraceMetal Grade, 67–69%, Thermo Fisher Scientific Inc., USA) and 3 mL of hydrogen peroxide (Merck, perhydrol for analysis, 30%, Merck Group, Germany) were added. The samples were allowed to react for 30 min. Then, 0.1 mL of HF (Merck, Suprapur, 40%, Merck Group, Germany) was added. The two-phase microwave-assisted digestion was carried out in a CEM Mars 5× microwave oven using a preinstalled digestion program following the EPA3052-standard: (1) ramp to 170 °C for 4 min 30 s; (2) ramp to 180 °C for 3 min 30 s; and (3) hold for 9 min 30 s. After the samples had cooled down, 1 mL of 4% boric acid (previously prepared) was added and a second microwave digestion phase was carried out using the following program: (1) ramp to 170 °C for 15 min and (2) hold for 10 min. Then, 0.5 mL of an internal standard mixture (Y, Sc, and Au) was added to each sample and the samples were diluted to 25 mL.

The analyses were carried out using Agilent 5110 VDV ICP-OES. To measure Si content, the data were taken from emission lines of 288.158 and 250.690 nm. A Sc wavelength of 361.383 nm was used as an internal standard. Quality control standards and blanks were used to ensure the reliability of the results.

### Statistical Analysis

Statistical significance (*p* < 0.05 marked with *, *p* < 0.01 as **, and *p* < 0.001 as ***) between groups was calculated using an unpaired two-tailed Student’s *t*-test.

## Results and Discussion

**Figure 1:**
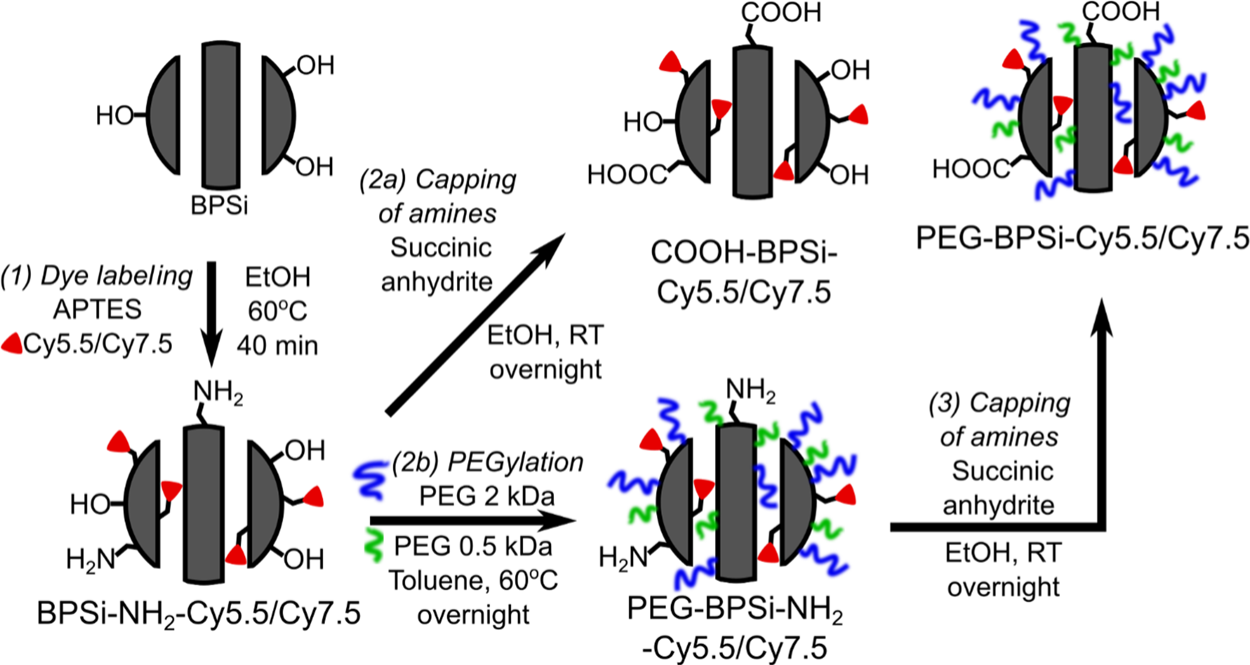
Scheme of BPSi-OH NP surface functionalization with cyanine 5.5 or 7.5 dyes and two differently sized PEG molecules. Free −NH_2_ groups were capped with succinic anhydrite to reduce ζ potential and improve colloidal stability.

The BPSi NPs produced by the reaction of NaSi with NH_4_Br under an inert atmosphere^24^ after treatment with HF and milling were predominantly covered by hydrophobic Si–H bonds. To enable further surface functionalization, the NPs were oxidized with HCl and hydrogen peroxide^27^ to obtain BPSi-OH NPs with surface −OH groups. BPSi-OH NPs were then functionalized with amine groups to graft Cy5.5 or Cy7.5 dyes and PEG of two sizes: 0.5 and 2 kDa (Figure 1). The use of two PEG sizes ensured efficient coverage to enhance biocompatibility and prolong colloidal stability upon systemic administration.^14,26^ The conjugation of the dye to APTES typically left many APTES amine groups unreacted, resulting in strong positive ζ-potentials even after PEG coating (Tables 1 and S1, Supporting Information). Positive ζ potential in turn led to fast aggregation even of the PEGylated NPs in PBS. Therefore, unreacted amine groups were capped with succinic anhydrite to switch ζ potential from positive to negative values, as depicted in Figure 1.

**Table 1:** Particle Diameters and ζ Potentials of BPSi NPs with Different Coatings^a^

Particles	size (nm)	PDI	ζ potential (mV)
BPSi-NH2-Cy7.5			
140	135 ± 34	0.101	51.3 ± 12.1
180	180 ± 63	0.096	42.7 ± 13.8
300	295 ± 73	0.124	42.6 ± 14.6
COOH-BPSi-Cy7.5			
140	80 ± 31	0.117	–50.6 ± 10.8
180	180 ± 63	0.098	–48.1 ± 10.3
300	286 ± 89	0.107	–49.9 ± 11.3
PEG-BPSi-Cy7.5			
140	140 ± 54	0.11	4.1 ± 6.7
200	195 ± 62	0.107	3.9 ± 6.5
300	308 ± 97	0.107	–4.6 ± 8.1

a *n* = 3, ±value is SD.

**Figure 2:**
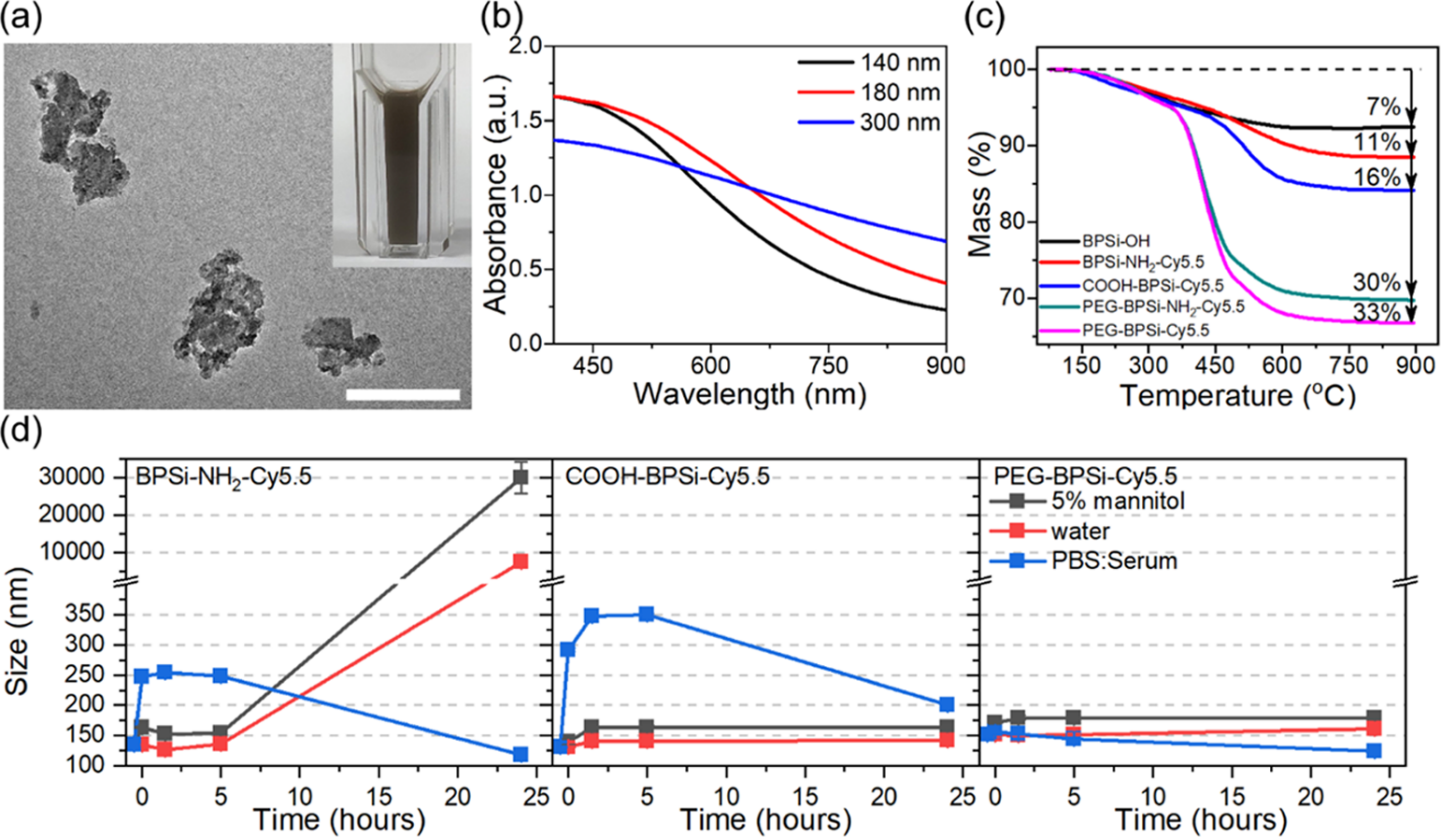
(a) Typical view of PEG-BPSi-Cy5.5 NPs before size separation. The scale bar is 200 nm. The inset shows a photograph of 0.2 mg/mL BPSi-OH dispersion. (b) UV–vis absorption of BPSi-OH NPs with different particle sizes. (c) Mass losses of BPSi-OH (black), BPSi-NH_2_-Cy5.5 (red), COOH-BPSi-Cy5.5 (blue), PEG-BPSi-NH_2_-Cy5.5 (green), and PEG-BPSi-Cy5.5 (magenta) measured by TGA. (d) Colloidal stability of 140 nm BPSi-NH_2_-Cy5.5, COOH-BPSi-Cy5.5, and PEG-BPSi-Cy5.5 in 5% mannitol solution (black), water (red), and PBS/serum 1:1 volume mixture (blue).

Representative TEM images of PEG-BPSi-Cy5.5 particles are shown in Figure 2a. NPs were prepared by ball milling of the larger microparticles obtained after the NaSi reduction, and they presented irregular shapes. The NP suspensions had dark color (Figure 2a) and they efficiently absorbed infrared radiation as published before, thus opening a possibility for efficient PTT.^24^ The size of the particles, however, affected the absorption of light (Figure 2b). The largest 300 nm particles absorbed light better in the red and infrared regions of the spectrum than their smaller counterparts. Since the pore sizes in BPSi are much smaller than the visible light wavelength, effective medium approximation can be used to evaluate optical properties of the NPs.^28^ Here, the NPs are described as a uniform solid with an effective dielectric permittivity constant calculated as the weighted sum of the dielectric permittivities of the NP material and the solvent in the pores. Therefore, higher absorption at long wavelengths is likely to be attributed to a longer optical path length in large particles. At short wavelengths, the absorption is limited to narrow nanometer-scale near-surface regions due to the band gap excitation, making the differences of light absorption between various sizes less-pronounced.

The surface of BPSi-OH NPs was first modified simultaneously with amine groups (APTES) and either Cy5.5 (for *in vitro*) or Cy7.5 (for *in vivo*) dyes. This resulted in additional TGA mass loss of 4.1 ± 0.2% compared to BPSi-OH NPs (Figure 2c). BPSi-NH_2_-Cy5.5 aggregated immediately in 5% mannitol solution and PBS/serum 1:1 mixture due to the positive charge of unreacted amine groups. In water, the aggregation was also observed for BPSi-NH_2_-Cy5.5 of all particle sizes after 24 h of incubation (Figures 2d and S1–S3, Supporting Information). Therefore, the unreacted amines were capped with succinic anhydrite, which effectively switched ζ potential to negative values and increased mass loss of COOH-BPSi-Cy5.5 by another 5.1 ± 0.2% compared to BPSi-NH_2_-Cy5.5 (Figure 2c). COOH-BPSi-Cy5.5 aggregated less than BPSi-NH_2_-Cy5.5 in water and 5% mannitol but still aggregated fast in PBS/serum. Thus, it is essential to further improve colloidal stability properties of the NPs for long systemic residence time. To realize this aim, BPSi-NH_2_-Cy5.5 was PEGylated with two different PEG molecules as it was previously demonstrated that such dual-PEG combination provides a longer blood circulation time than the mono-PEGylation.^14^ PEG coating added 19.2 ± 0.4% of mass loss in TGA measurement compared to that from BPSi-NH_2_-Cy5.5. Following PEGylation, unreacted amine groups were capped with succinic anhydrite to obtain final PEG-BPSi-Cy5.5 (or Cy 7.5) NPs (Figure 2c). PEG-BPSi-Cy5.5 was the most stable in the PBS/serum mixture; after 24 h of incubation, it did not show any aggregation (Figures 2d and S1–S3, Supporting Information).

The aggregation of NPs was further studied with TEM (Figures S4–S6, Supporting Information) after 4 h of incubation at 37 °C in 50 mM PBS. As shown in Supporting Information, BPSi-NH_2_-Cy5.5 NPs were heavily aggregated into large clusters after 4 h. COOH-BPSi-Cy5.5 NPs aggregated somewhat less and relatively smaller clusters than for BPSi-NH_2_-Cy5.5 NPs were found together with separate NPs. PEG-BPSi-Cy5.5 NPs showed no sign of aggregation and remained spread as separate NPs across the TEM grid. Overall, the results from TEM images are consistent with those shown in Figure 2. The positive charge of BPSi-NH_2_-Cy5.5 NPs induced the most rapid aggregation and the negative charge of COOH-BPSi-Cy5.5 NPs decreased the rate of agglomeration but could not fully prevent it, while PEG coating combined with COOH– capping efficiently suppressed aggregation and kept NPs separated from each other.

**Figure 3:**
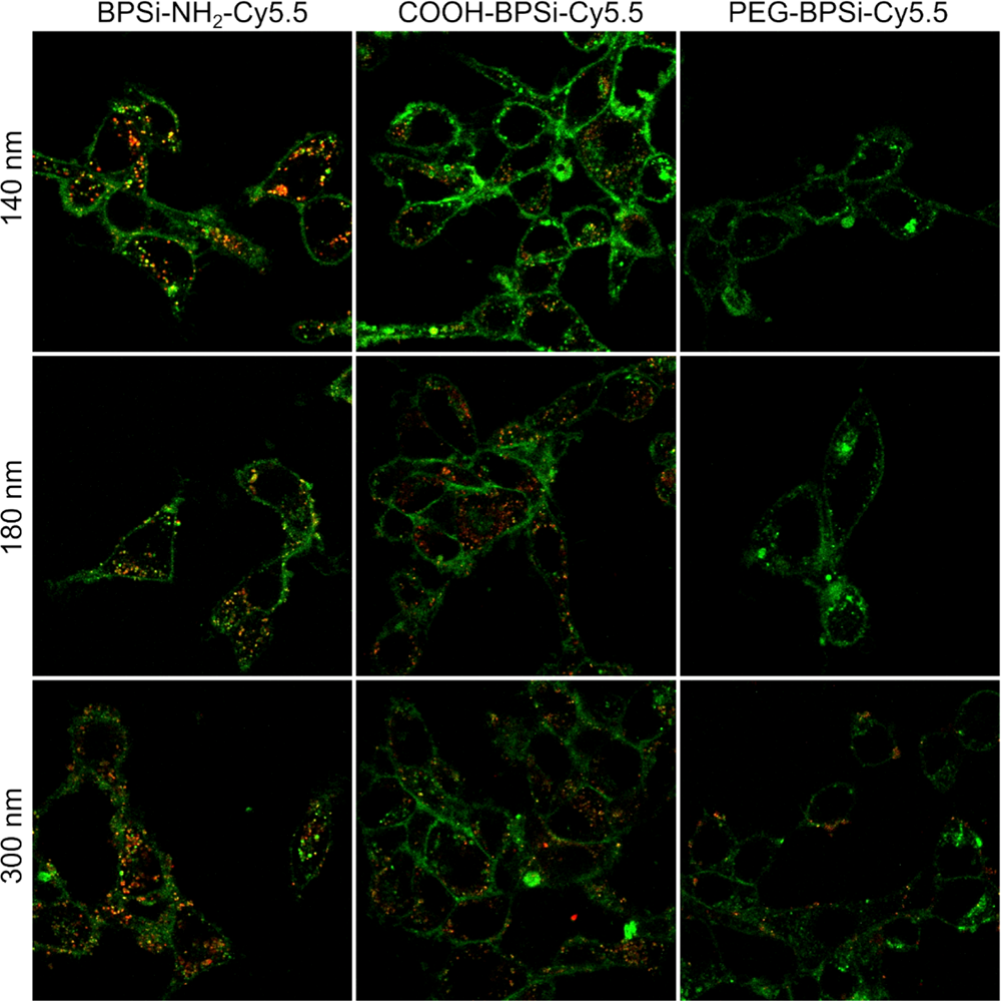
Internalization of BPSi-NH_2_-Cy5.5, COOH-BPSi-Cy5.5, and PEG-BPSi-Cy5.5 NPs of different sizes into CT26 cells after 24 h of incubation. Cell membranes are shown in green, and NPs are shown in red.

Depending on the surface coating, BPSi NPs were internalized differently by cells with similar patterns observed in both CT26 and RAW 264.7 cells. In addition to fast aggregation and subsequent precipitation on cells, positively charged BPSi-NH_2_-Cy5.5 NPs were electrostatically attracted to negatively charged cell membranes. Large aggregates of BPSi-NH_2_-Cy5.5 NPs were observed by TEM both outside the cells and internalized (Figure S14, Supporting Information). The positive charge resulted in higher internalization into CT26 (Figures 3 and S7–S9, Supporting Information) and RAW 264.7 (Figures S10–S12, Supporting Information), compared to negatively charged COOH-BPSi-Cy5.5 and neutral PEG-BPSi-Cy5.5 NPs. Such a high internalization of BPSi-NH_2_-Cy5.5 NPs was relatively independent of the particle size (Figure 3). Negatively charged COOH-BPSi-Cy5.5 NPs had electrostatic repulsion with cell membranes while still appeared aggregated in cell medium but to a lesser extent than BPSi-NH_2_-Cy5.5 NPs. Only few internalized aggregates of COOH-BPSi-Cy5.5 NPs were found by TEM, and no NPs were located on the cell membrane (Figure S12, Supporting Information). For COOH-BPSi-Cy5.5 NPs, the size-dependent cellular internalization was observed: large particles were internalized better than small ones. The observed trend was attributed to the experimental conditions, where larger NPs have larger mass per particle, and thus, they are faster adhered to cell membranes by gravity than smaller NPs. The PEG coating effectively prevented particles from aggregation. Only few large 300 nm PEG-BPSi-Cy5.5 were found to be internalized into CT26 and RAW 264.7 cells possibly due to gravitational force that governed cell membrane adsorption. The decreased interaction with the cell membrane of 140 and 200 nm PEG-BPSi-Cy5.5 effectively reduced their internalization; no internalized 140 nm NPs were found using TEM (Figure S13, Supporting Information).

**Figure 4:**
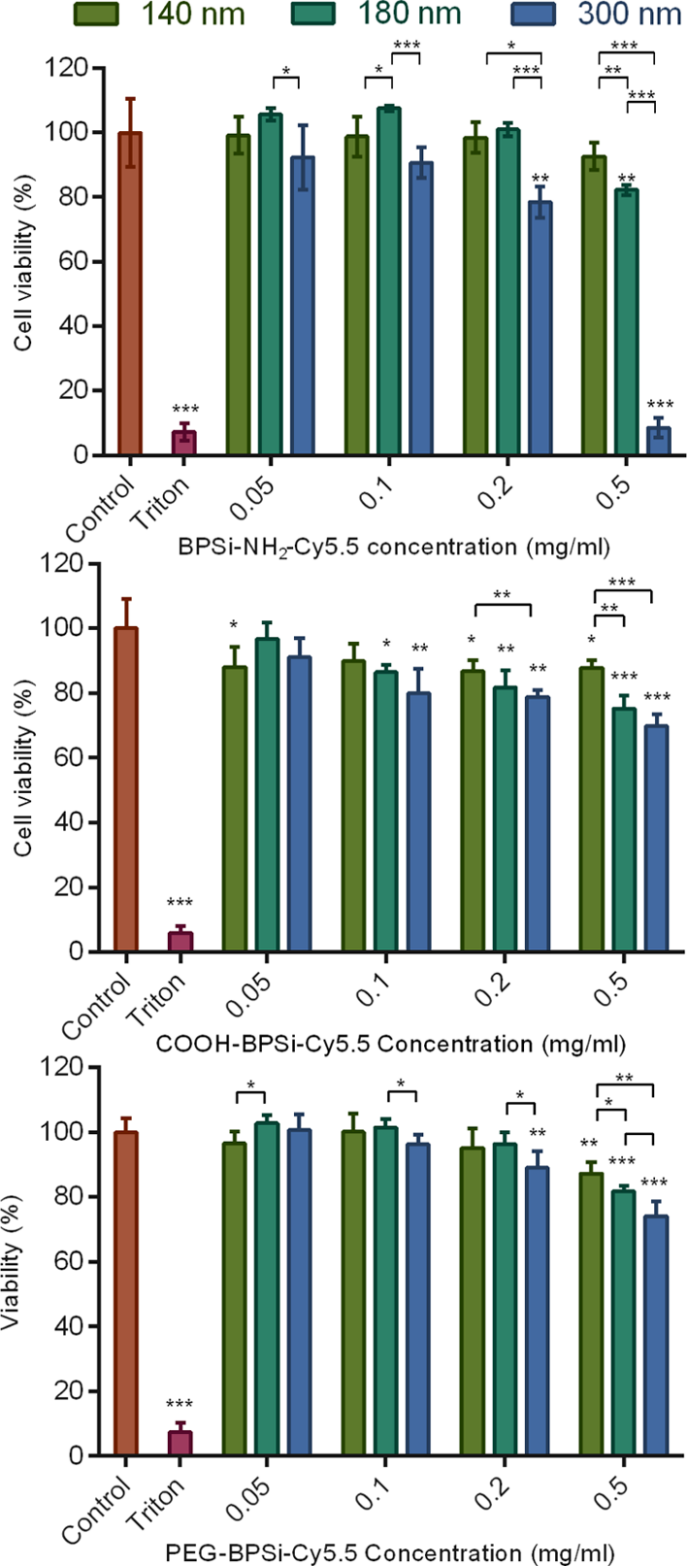
Viability of CT26 cells after incubation with BPSi-NH_2_-Cy5.5, COOH-BPSi-Cy5.5, and PEG-BPSi-Cy5.5 NPs of different sizes (mean ± standard deviation, *n* = 4; significance analysis *p < 0.05, **p < 0.01, and ***p < 0.001).

In accordance with cell internalization, both sizes and surface coatings of BPSi NPs affected cell viability (Figure 4). The lowest cell viabilities were found for positively charged 300 nm BPSi-NH_2_-Cy5.5 at 0.2 and 0.5 mg/mL concentrations. These NPs internalized the most into cells and thus impaired their functions accordingly at high concentrations, which eventually led to cell death. In general, the large 300 nm NPs decreased cell viability the most for all studied surface coatings. The decrease was likely to be attributed to the gravitational force of the NPs in the 2D cell culture system, in which 300 nm particles adhered more on the cells than the smaller ones and thus impaired cell viability the most. Low and moderate concentrations up to 0.2 mg/mL were found to be generally safe independent of the coating. Only BPSi-NH_2_-Cy5.5 NPs resulted in cell viability slightly below 80%, while none of the other particles decreased cell viability by more than 20%, demonstrating good biocompatibility and low toxicity of BPSi NPs.

**Figure 5:**
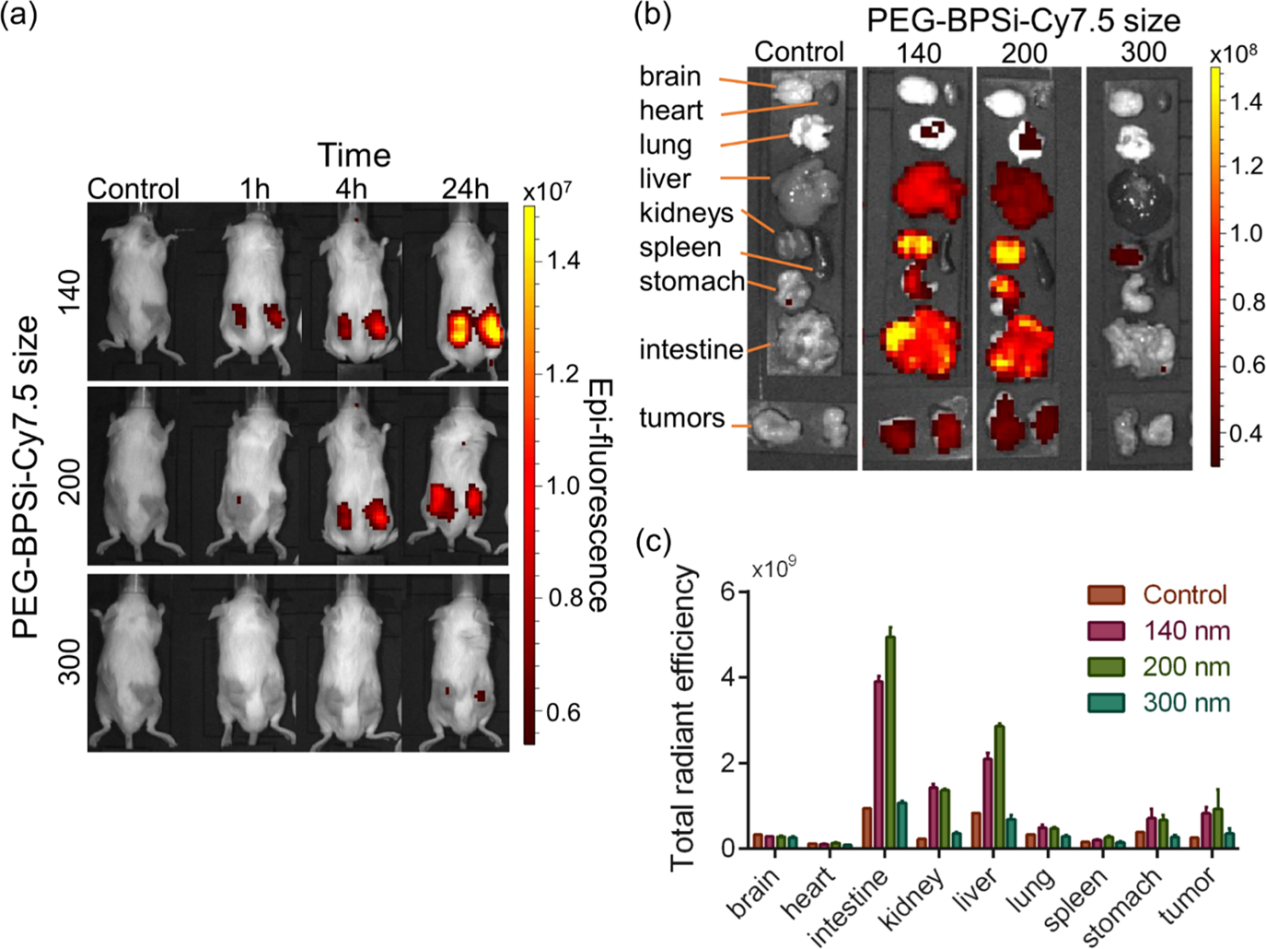
Biodistribution of PEG-BPSi-Cy7.5 NPs of different sizes using IVIS after intravenous administration. The injection dose was 5 mg of NPs per animal. (a) Dorsal view depicting the fluorescence signal from tumors at 1, 4, and 24 h time points. (b) *Ex vivo* fluorescence of organs and tumors taken 24 h after injection. (c) Total radiant efficiency calculated from *ex vivo* fluorescence after selecting appropriate ROIs using IVIS software. *N* = 3.

For *in vivo* imaging of the organ distribution profiles, NPs were conjugated with Cy7.5 instead of Cy5.5. Cy7.5 dye has the excitation/emission profiles in the near-infrared part of the spectrum; thus, it is more suitable for *in vivo* imaging than Cy5.5. Since Cy7.5 and Cy5.5 are from the same category and have the same functional group, the change in the dye to Cy7.5 is not expected to significantly affect physicochemical properties of the labeled NPs. PEG-BPSi-Cy7.5 NPs of the size of 140, 200, and 300 nm were intravenously injected into CT26 tumor-bearing mice at a dose of 5 mg/animal. At such a high dose, no obvious toxicity was observed as animals behaved normally without weight loss up to 2 weeks postinjection (data not shown). Based on the whole-body imaging from dorsal view (Figures 5a and S17, Supporting Information), it can be seen that PEG-BPSi-Cy7.5 NPs of smaller sizes tended to accumulate better in tumors. The fluorescence intensity was the highest for 140 nm NPs and the lowest for 300 nm NPs with the signals increasing over time. The same trend was observed by *ex vivo* imaging on excised tumors and other organs (Figure 5b). In addition, high fluorescent signals were observed in the liver, intestine, kidneys, and stomach. There were some fluorescence signals detected in lungs, but no signals were measured in the spleen. One can notice that there was almost no fluorescence captured for tissues from the animals treated with 300 nm NPs despite the “visible” uptake of NPs in certain tissues such as the liver (e.g., the much darker color of the liver compared to the control liver) (Figure 5b). As demonstrated in Figure 2b, 300 nm NPs absorbed approximately 180 and 80% more light in the near-infrared region than 140 and 200 nm NPs, respectively. Thus, both excitation radiation and light emitted by Cy7.5 were likely to be attenuated by large NPs to a greater extent than by the smaller counterparts (Figure S19, Supporting Information). Possible aggregation of NPs formed in tissues with higher uptake, such as the liver and spleen, may further increase the absorption of light and decrease the emitted fluorescence signals. Therefore, the calculated total radiance efficiency from different organs and tumors (Figure 5c) could not provide an adequate assessment on the organ biodistribution of the studied BPSi NPs.

There are two other difficulties in addition to light absorption by the NPs. The first one is the varied thickness of the organs; the fluorescence signal from the organs may come only from the parts close to the surface, while deeper light penetration is limited by the absorption in tissues and the NPs. The second one is the fact that total radiance efficiency is proportional both to the area of ROI and to the fluorescence signal in each pixel of the ROI. Thus, some organs with a big ROI and low background fluorescence can have higher total radiance efficiency than organs with a small ROI but somewhat higher fluorescence. As one can see from Figure 5c, the brain and heart have fluorescence similar to the background and control animal, but the total radiance efficiency is higher for the brain than for the heart due to higher ROI area.

Despite the discussed drawbacks of IVIS, qualitative assessment can still be carried out based on the data from 140 to 200 nm PEG-BPSi-Cy7.5 NPs (Figure 5b,c). Clearly, part of the NPs eventually accumulated in the liver and spleen, which are the organs responsible for eliminating alien bodies from systemic circulation. Interestingly, high fluorescence was observed from the stomach, intestine, and kidney. The signals from these tissues may be a result of some free dyes dissociated from the NPs. It is also well-known that NPs with ∼100 nm can experience renal excretion. In mice injected with free Cy7.5, *ex vivo* images indicate that Cy7.5 accumulated in most of the assessed tissues with a preferential accumulation in the liver (Figure S21, Supporting Information). In this regard, the exceptionally high fluorescence signals in the stomach and intestine are more likely attributed to the PEG-BPSi-Cy7.5 NPs that underwent the hepatobiliary clearance pathway,^29−31^ where the NPs are transported from the liver to the stomach and small intestine and can be finally removed from the body by fecal excretion, as reported in other studies.

**Figure 6:**
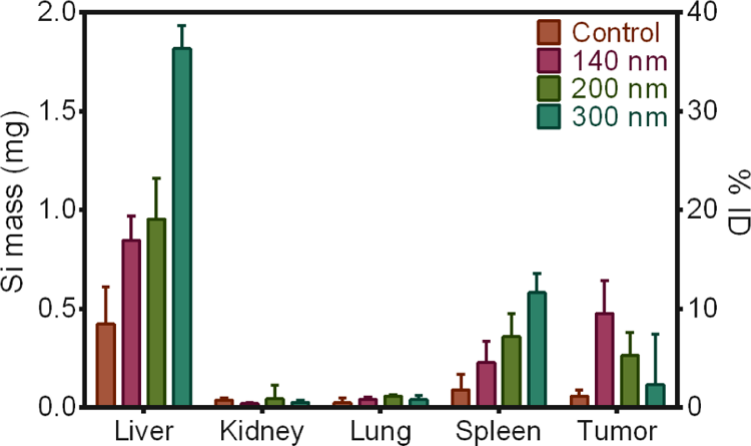
Biodistribution of PEG-BPSi-Cy7.5 NPs of different sizes based on Si content analysis with ICP-OES. Left and right axes show the total mass of Si in mg in each organ and percent of injected dose (% ID, ID = 5 mg), respectively. *N* = 3.

Precise quantification of Si content in major organs and tumors was further performed using ICP-OES (Figures 6 and S22, Supporting Information). Clearly, the liver and spleen were found to be the two organs with the highest uptake of PEG-BPSi-Cy7.5 NPs. The 300 nm NPs accumulated the most in the liver, while 140 and 200 nm NPs showed lower but similar uptake in the liver. A similar trend was observed in the spleen. Thus, the increase in NP size resulted in enhanced accumulation in the liver and spleen, known reservoirs of NPs after systemic administration.^32−34^ Interestingly, despite the observed high fluorescence intensity in kidneys, no Si content was detected, suggesting the possible release of Cy7.5 from the NPs. In contrast to the liver and spleen, the smallest 140 nm PEG-BPSi-Cy7.5 showed the highest tumor uptake, achieving 9.5 ± 3.4% of the injected dose (ID) being retained in tumors (3.5 ± 1.7% ID/g of tumor). With the increase in NP size, less uptake in tumors was detected: 5.3 ± 2.3% ID/g and 2.3 ± 5.1% ID/g for 200 and 300 nm NPs, respectively. Overall, the results demonstrated that the particle size is critical for passive tumor targeting and the small PEG-BPSi NPs with a size of 140 nm in diameter showed efficient accumulation in tumors.

**Figure 7:**
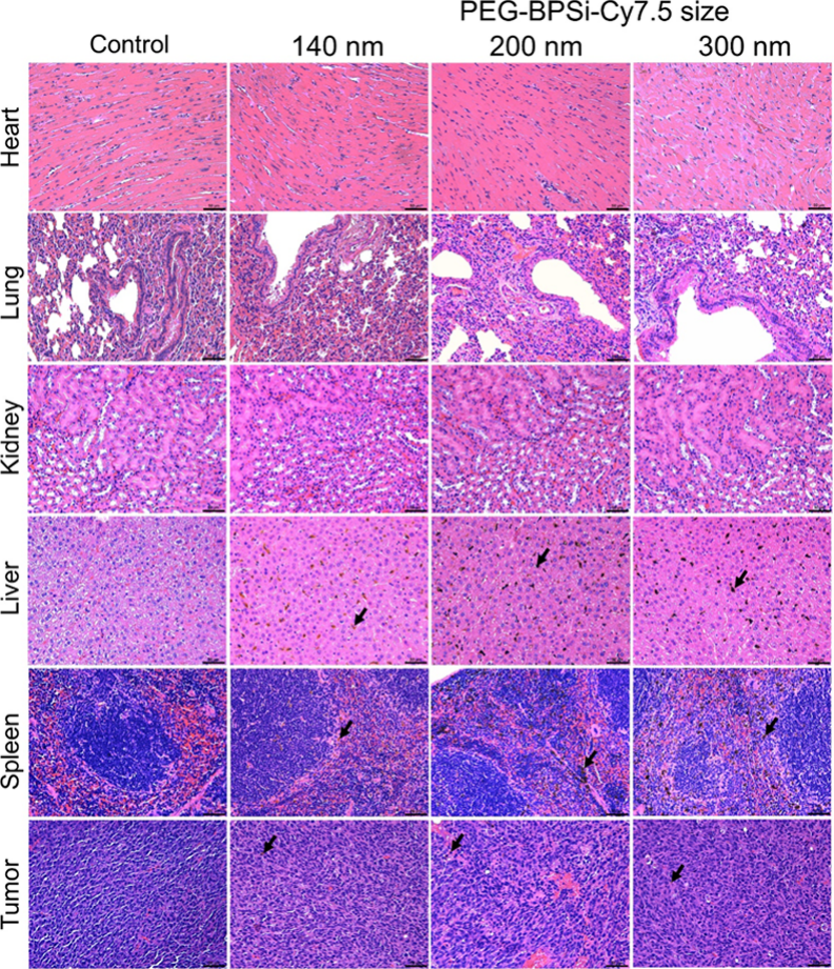
Histological examination of vital organs with H&E staining. Arrows point to PEG-BPSi-Cy7.5 patches in the spleen, liver, and tumor tissues. The scale bar is 50 μm.

Histological assessments of organs and tumors (Figures 7 and S23, Supporting Information) supported the findings from ICP-OES analysis. Numerous PEG-BPSi-Cy7.5 NP patches were clearly visualized in the liver and spleen, possibly taken up by the abundant resident macrophages in these two organs. The accumulation of PEG-BPSi-Cy7.5 in tumors was less visible in comparison, indicating that the PEG-BPSi NPs may be distributed more evenly in tumors. Uptake of PEG-BPSi NPs by Kupffer cells in the liver was also observed. The large patch sizes in the liver and spleen are expected to attenuate light to an extent that both the excitation and emission radiations are hindered by high absorbance in the patches.

## Conclusions

BPSi NPs are efficient absorbers of infrared radiation. This property makes them an excellent candidate for PTT of cancer, while their porosity makes it possible to load them with chemotherapeutic drugs opening a way for a synergistic therapy. In order to achieve sufficiently high heating to induce cell death, a high number of particles must reach the tumor. Herein, we examined the influence of size and coating of BPSi NPs on their *in vitro* and *in vivo* behaviors. BPSi NPs without PEG coating aggregated fast *in vitro*, adhered to the cell membrane and internalized into cells. The adherence and subsequent internalization were pronounced more for positively charged 300 nm NPs than that for their smaller and negatively charged counterparts. PEG coating further improved biocompatibility of the NPs, demonstrating no toxicity up to 1 mg/mL concentration and no aggregation up to 24 h in the PBS/serum mixture. Accordingly, biodistribution of PEG-coated BPSi NPs in tumor-bearing mice was studied. Intrinsic strong light-absorption properties of BPSi NPs limited the assessment of their biodistribution by optical imaging. Instead, the ICP-OES method, based on elemental Si analysis, successfully quantified the uptake of BPSi NPs in tissues and tumors. Most of the BPSi NPs were found to accumulate in the liver and spleen. With the decrease in particle size from 300 to 140 nm, their accumulation in tumor increased. Nearly 10% injected dose in tumors was achieved for 140 nm PEG-BPSi-Cy7.5 NPs, equivalent to 0.18 mg/g of tumor. This amount is known to generate sufficient heat for PTT to induce tumor cell death.

## Supporting Information Available

Additional characterization of the NPs with different surface modifications, including ζ-potential, colloidal stability, *in vitro* cell internalization into RAW 264.7 and CT26 cells, IVIS and ICP-OES measurements, and histologically stained with neutral red staining (PDF)
