# Supplementary material for: Comparison between Fluorescence Imaging and Elemental Analysis to Determine Biodistribution of Inorganic Nanoparticles with Strong Light Absorption
Source: ACS Appl Mater Interfaces. 2021 Aug 16;13(34):40392–400. doi: 10.1021/acsami.1c11875 (PMC8414481; doi:10.1021/acsami.1c11875)
Supplement: Supplementary file 1 — am1c11875_si_001.pdf [file am1c11875_si_001.pdf]

Supporting Information

Comparison between fluorescence imaging and elemental analysis to determine biodistribution of inorganic nanoparticles with strong light absorption

Konstantin Tamarov,[1] Julie Tzu-Wen Wang,[2] Juuso Kari,[1] Emilia Happonen,[1] Ilkka Vesavaara,[3] Matti Niemelä,[3] Paavo Perämäki,[3] Khuloud T. Al-Jamal,[2] Wujun Xu,[1,] and Vesa-Pekka Lehto[1]*

[1]Department of Applied Physics, Faculty of Science and Forestry, University of Eastern Finland, Kuopio 70211, Finland

[2]School of Cancer and Pharmaceutical Sciences, Faculty of Life Sciences & Medicine, King's College London, London SE1 9NH, UK

[3]Research Unit of Sustainable Chemistry, University of Oulu, Oulu 90570, Finland

*Corresponding author:

wujun.xu@uef.fi,

khuloud.al-jamal@kcl.ac.uk

Table S1. Zeta potentials of BPSi NPs with different coatings.

	In water, mV	In PBS, mV
BPSi-OH	-24.4 ± 0.9	-16.3 ± 1.5
BPSi-NH_2	32.2 ± 0.7	3.8 ± 1.1
BPSi-NH_2-Cy5.5	32.5 ± 0.7	6.8 ± 0.3
COOH-BPSi-Cy5.5	18.5 ± 0.6	-11.1 ± 0.7
PEG-BPSi-NH_2-Cy5.5	25.6 ± 0.9	3.0 ± 0.8
PEG-BPSi-Cy5.5	-7.6 ± 0.4	-1.4 ± 0.6

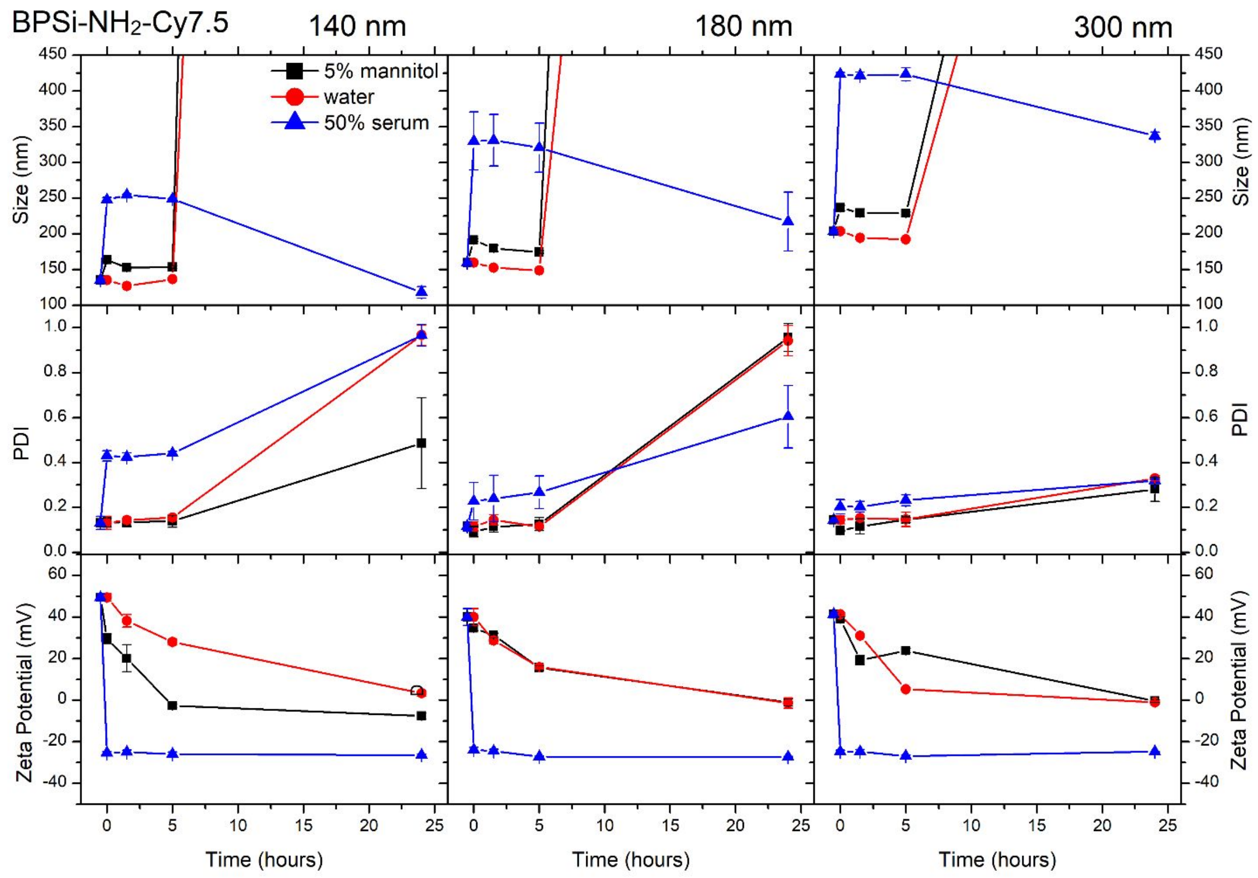

Figure S1. Colloidal stability of BPSi-Cy7.5 nanoparticles in 5% mannitol solution (back squares). water (red circles) and 50% serum (blue triangles). Columns show the stability for different article sizes: 140, 180 and 300 nm. Rows shows the change of size, polydispersity index (PDI) and zeta potential during the incubation at 37 °C.

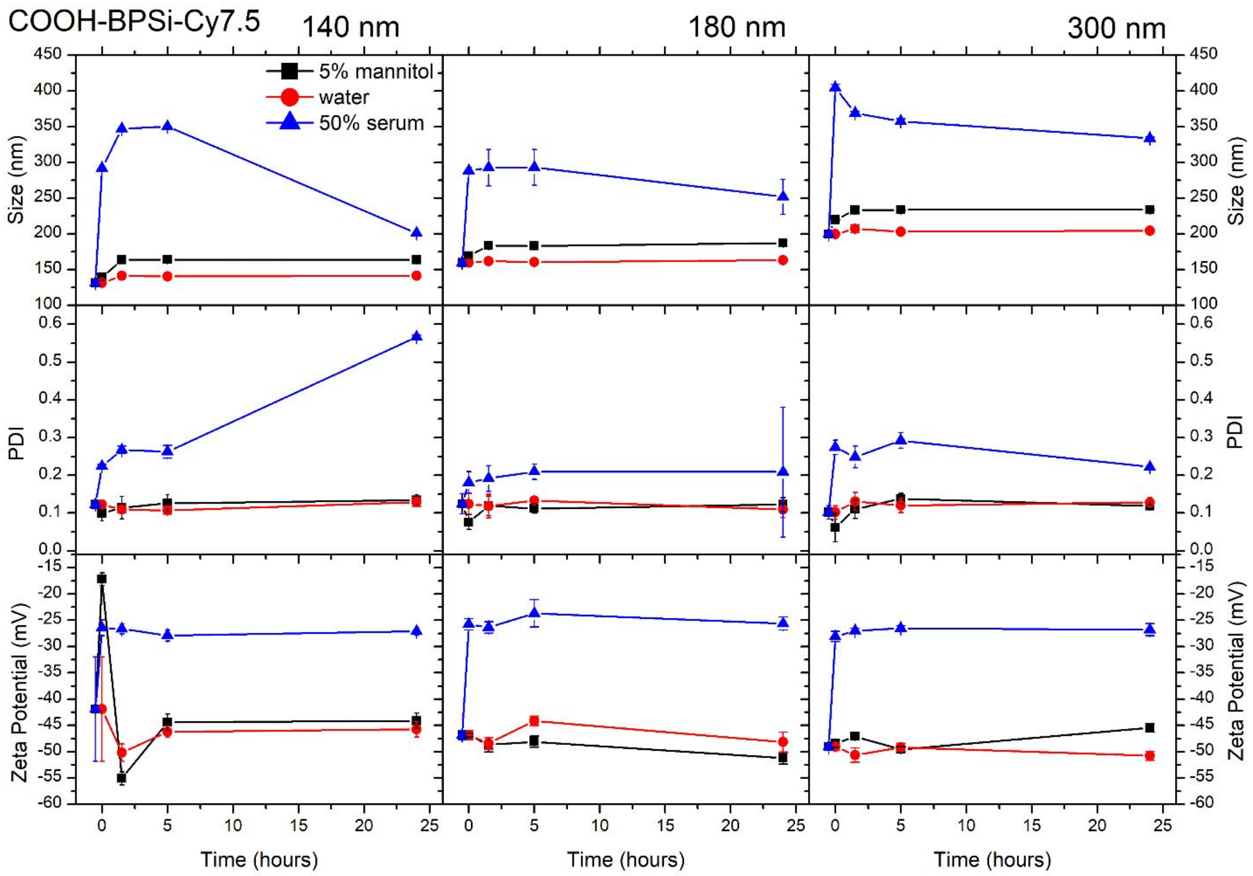

Figure S2. Colloidal stability of COOH-BPSi-Cy7.5 nanoparticles in 5% mannitol solution (back squares). water (red circles) and 50% serum (blue triangles). Columns show the stability for different article sizes: 140, 180 and 300 nm. Rows shows the change of size, polydispersity index (PDI) and zeta potential during the incubation at 37 °C.

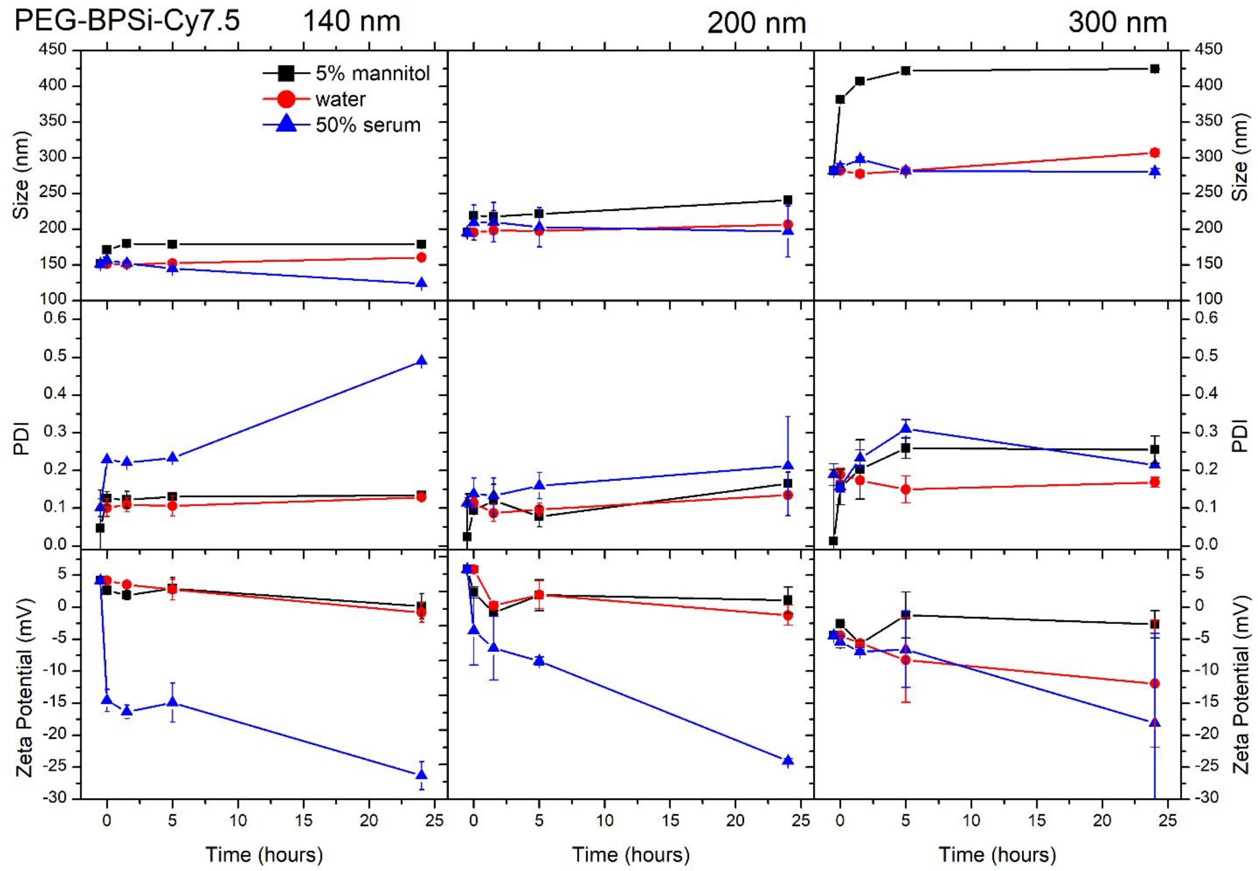

Figure S3. Colloidal stability of PEG-BPSi-Cy7.5 nanoparticles in 5% mannitol solution (back squares). water (red circles) and 50% serum (blue triangles). Columns show the stability for different article sizes: 140, 200 and 300 nm. Rows shows the change of size, polydispersity index (PDI) and zeta potential during the incubation at 37 °C.

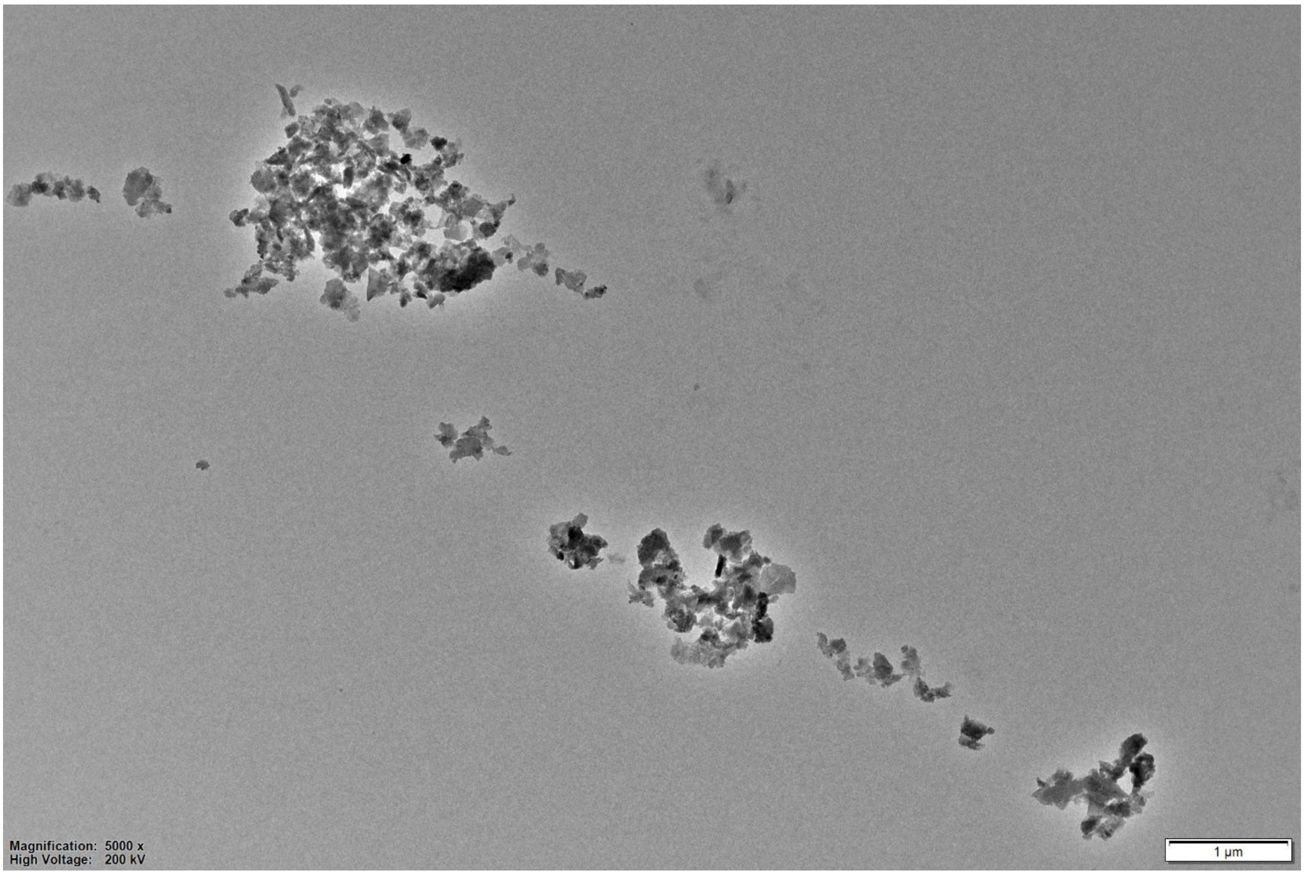

Figure S4. Aggregates of BPSi-NH_2 NPs after 4 h incubation at 37ºC in 50 mM PBS solution.

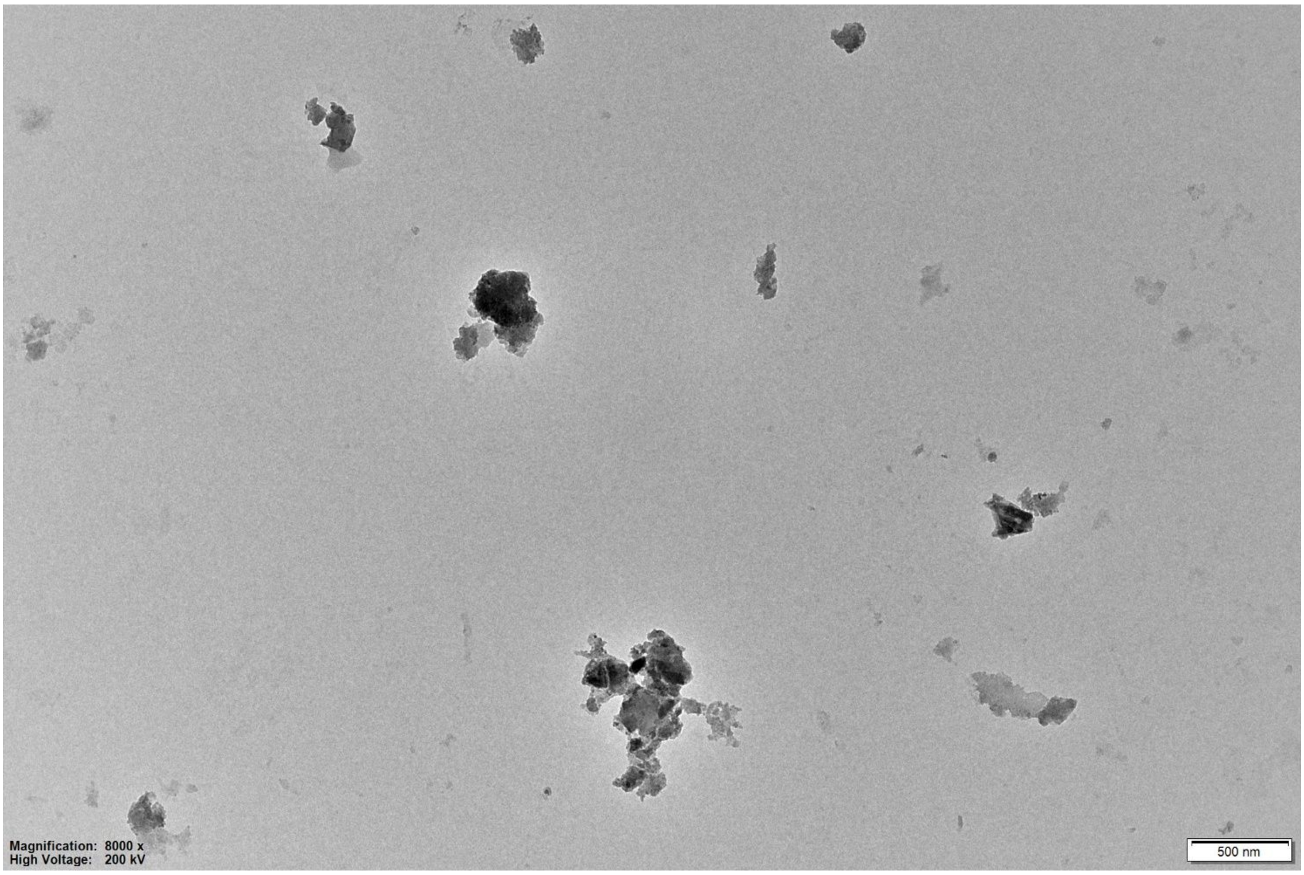

Figure S5. Aggregates and separate COOH-BPSi NPs after 4 h incubation at 37ºC in 50 mM PBS solution. Less aggregates were found, and they were smaller than in case of BPSi-NH_2-Cy5.5 NPs.

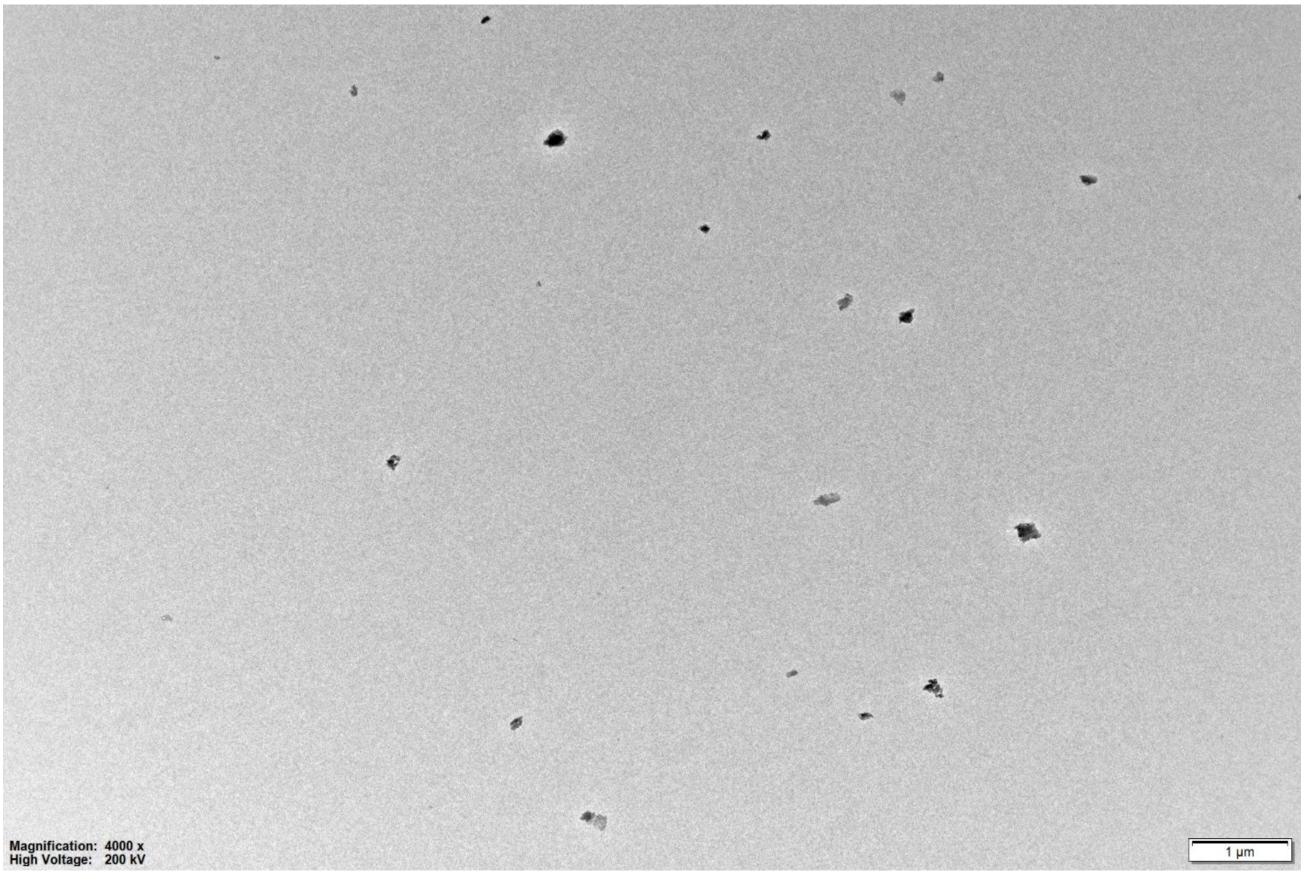

Figure S6. PEG-BPSi-Cy5.5 NPs after 4 h incubation at 37ºC in 50 mM PBS solution. Only separately located NPs were found.

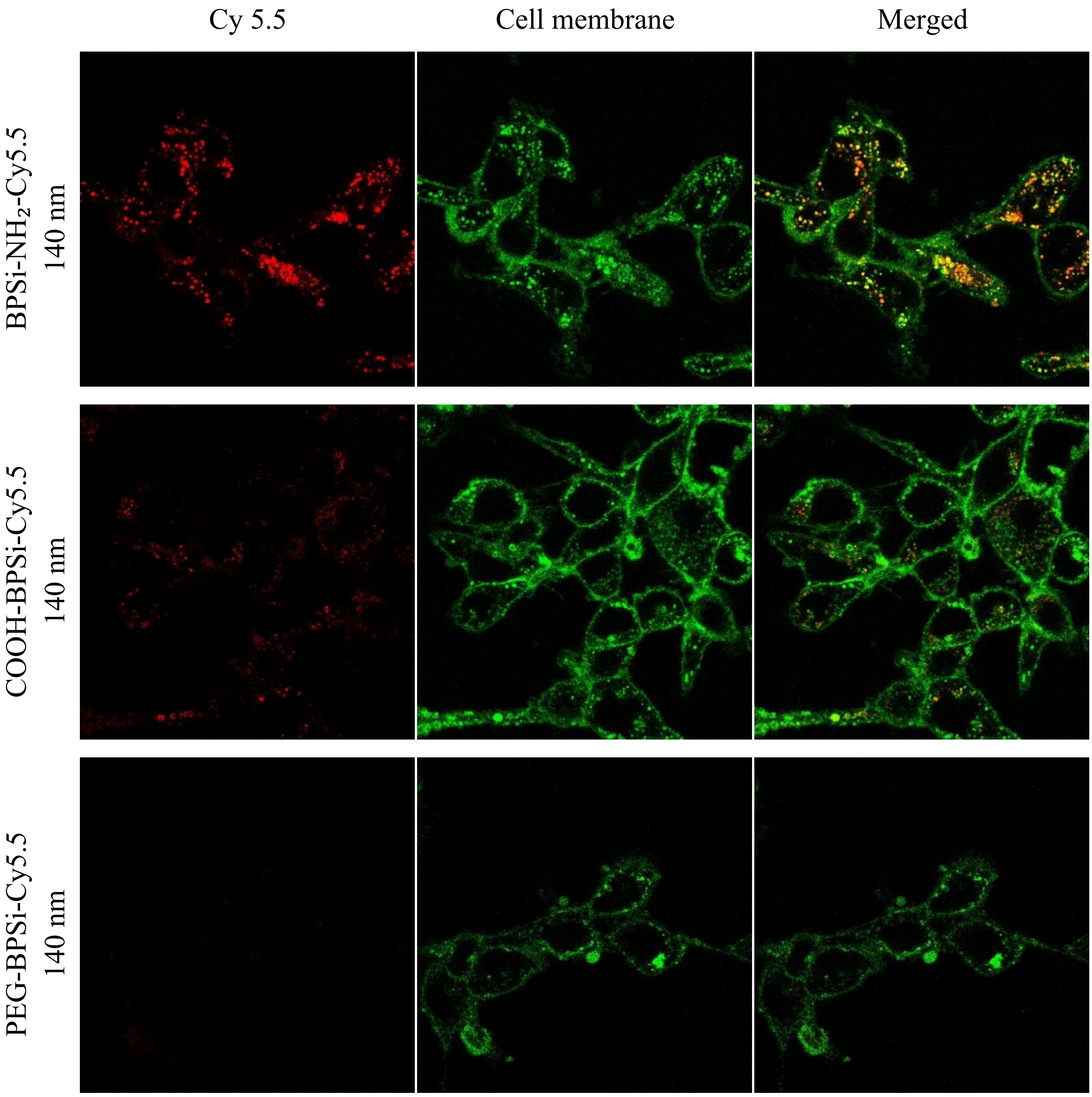

Figure S7. Internalization of 140 nm BPSi nanoparticles with different surface modifications by CT26 cells. Surface coatings are described in the Materials and Methods section. Left column: fluorescence from Cy5.5 conjugated to the particles. Middle column: cell membrane stained with CellMask. Right column: merged left and middle columns.

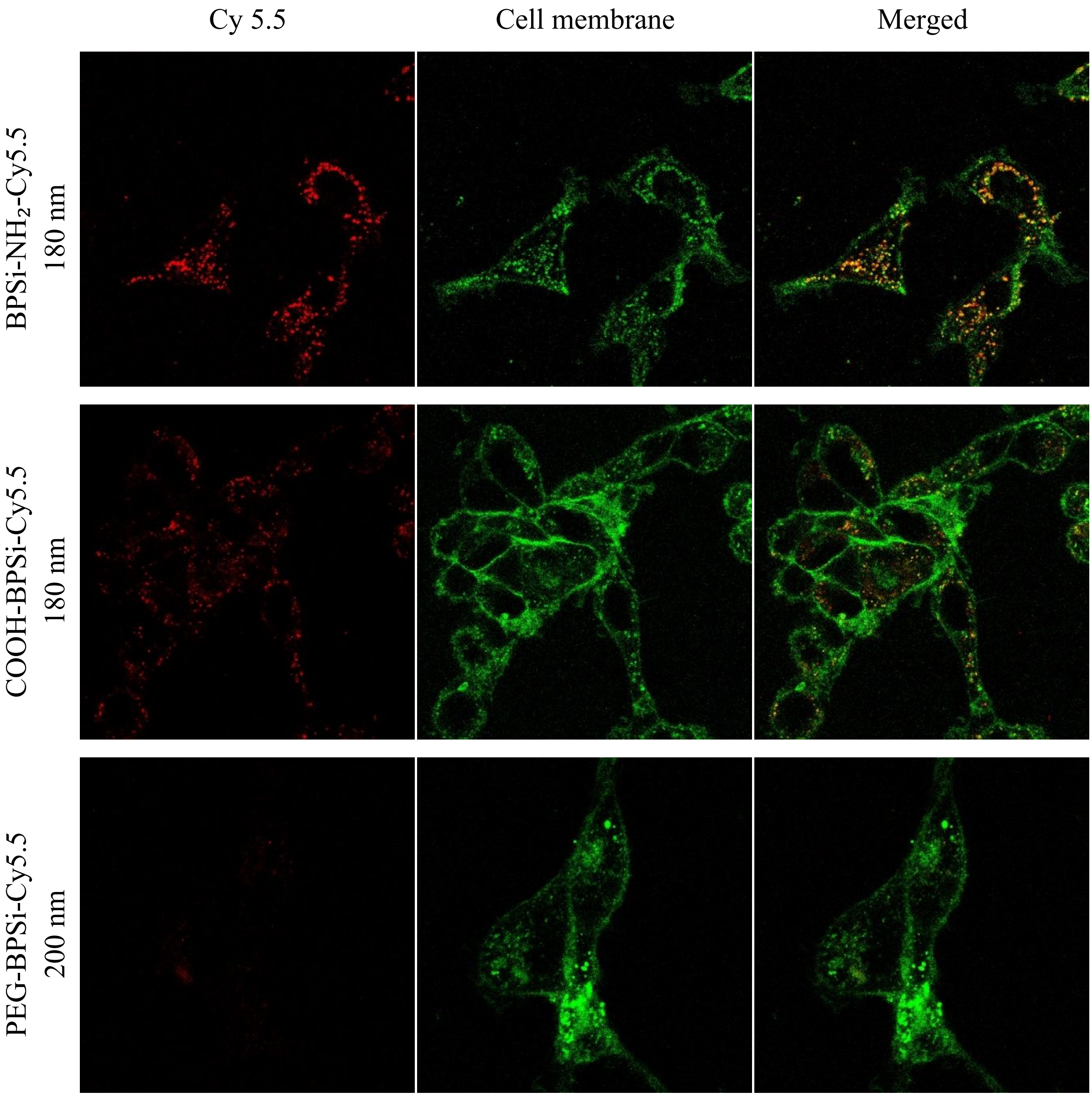

Figure S8. Internalization of 180 nm BPSi nanoparticles with different surface modifications by CT26 cells. Surface coatings are described in the Materials and Methods section. Left column: fluorescence from Cy5.5 conjugated to the particles. Middle column: cell membrane stained with CellMask. Right column: merged left and middle columns.

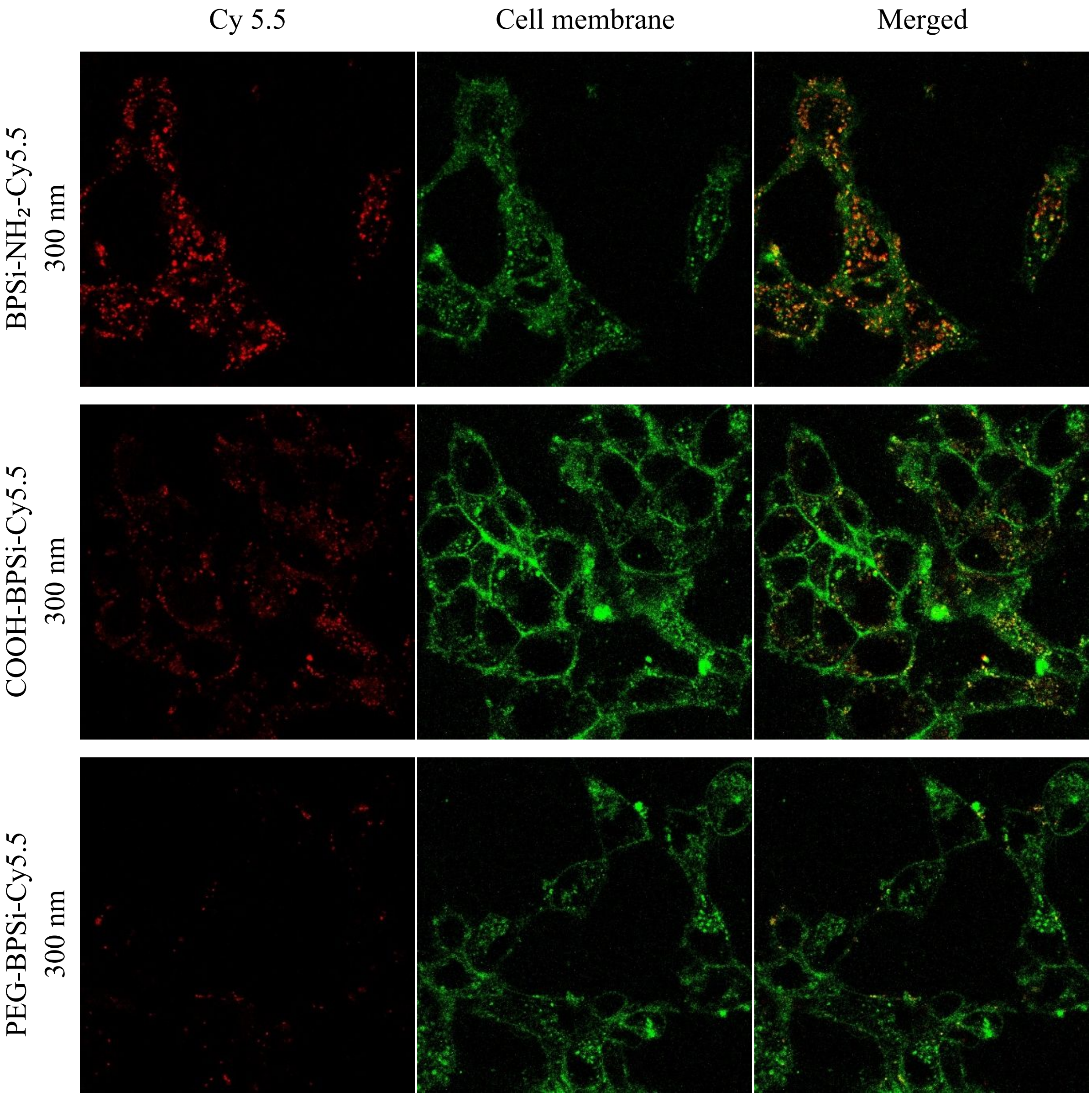

Figure S9. Internalization of 300 nm BPSi nanoparticles with different surface modifications by CT26 cells. Surface coatings are described in the Materials and Methods section. Left column: fluorescence from Cy5.5 conjugated to the particles. Middle column: cell membrane stained with CellMask. Right column: merged left and middle columns.

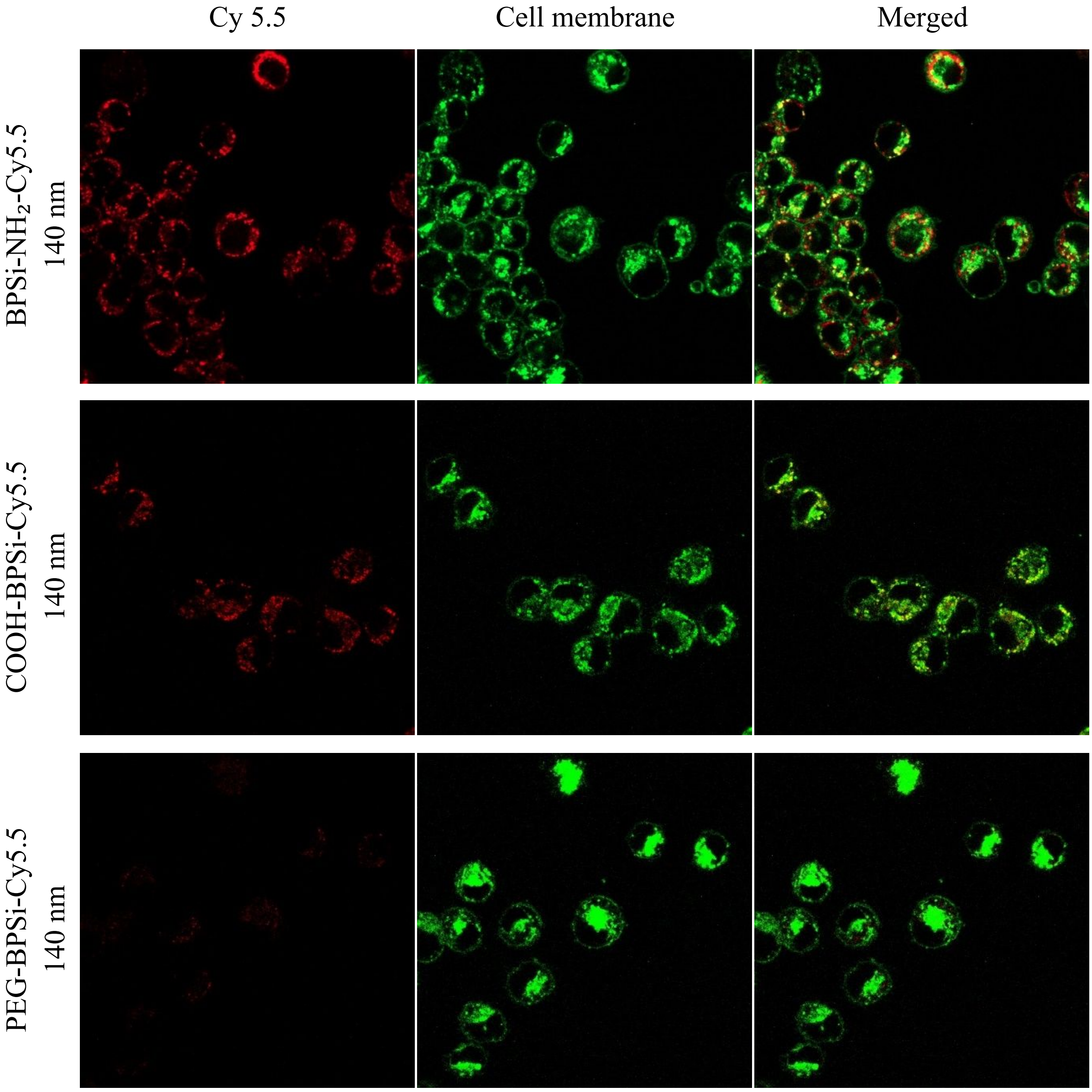

Figure S10. Internalization of 140 nm BPSi nanoparticles with different surface modifications by RAW 264.7 macrophages. Surface coatings are described in the Materials and Methods section. Left column: fluorescence from Cy5.5 conjugated to the particles. Middle column: cell membrane stained with CellMask. Right column: merged left and middle columns.

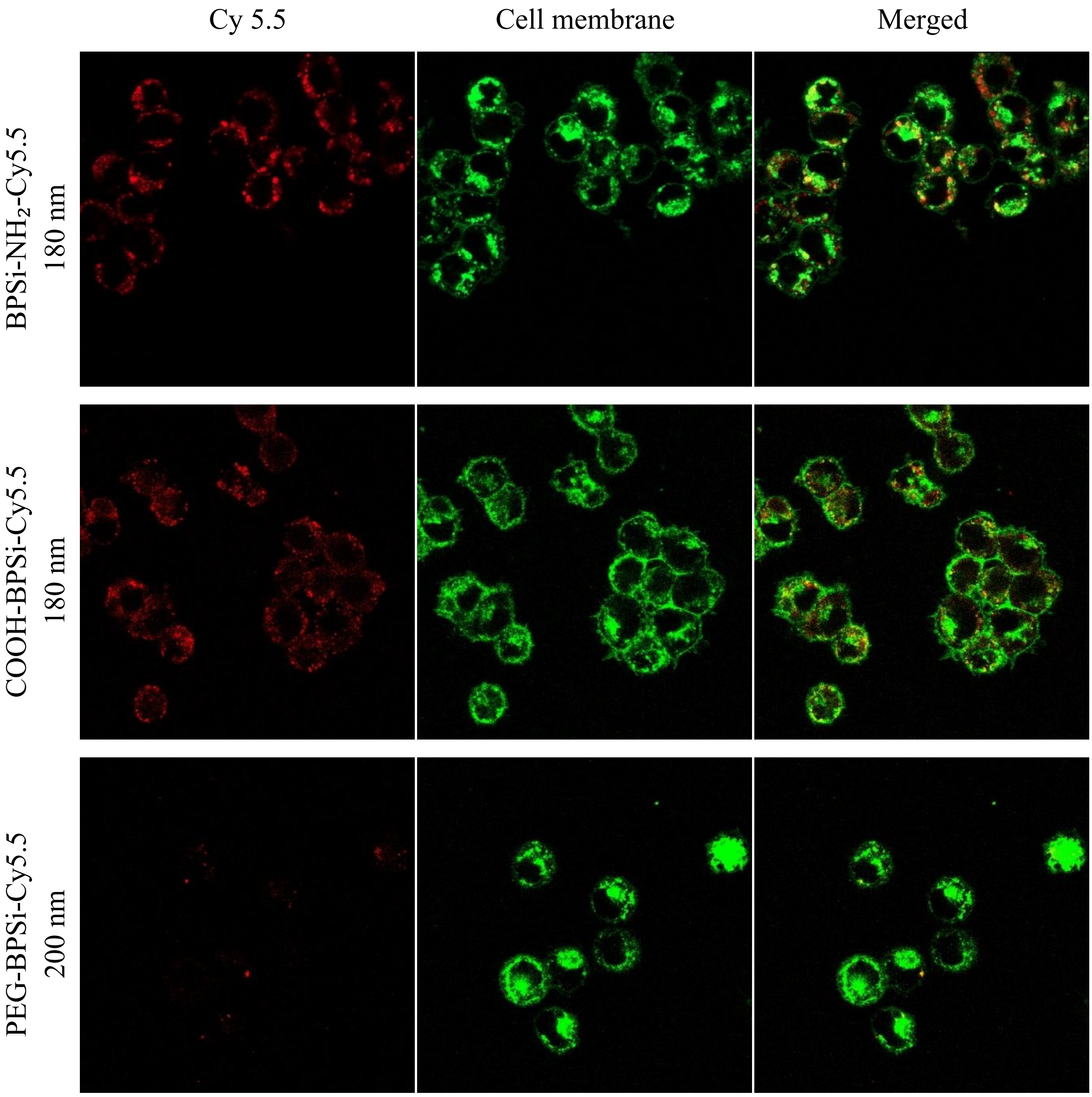

Figure S11. Internalization of 180 nm BPSi nanoparticles with different surface modifications by RAW 264.7 macrophages. Surface coatings are described in the Materials and Methods section. Left column: fluorescence from Cy5.5 conjugated to the particles. Middle column: cell membrane stained with CellMask. Right column: merged left and middle columns.

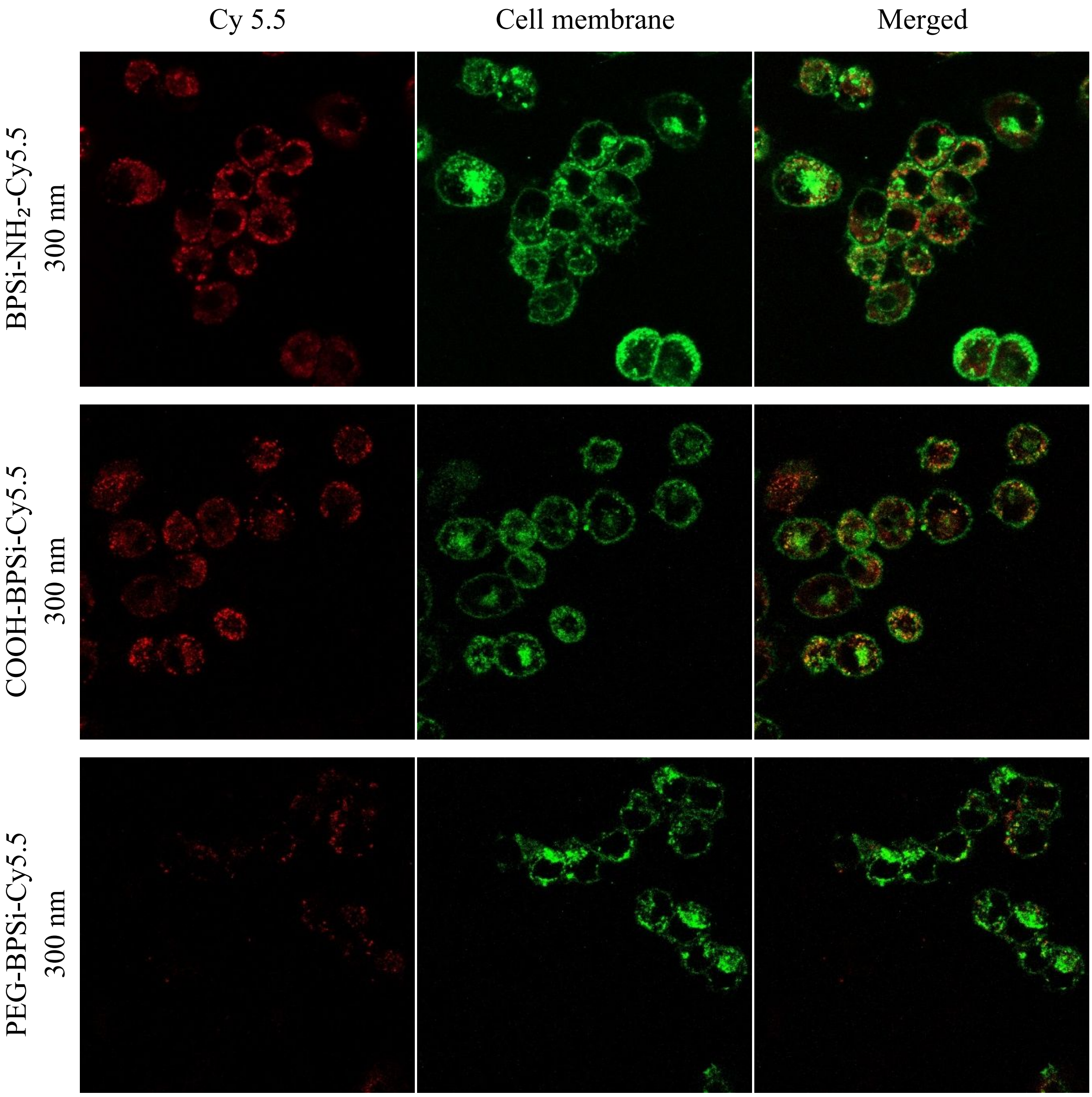

Figure S12. Internalization of 300 nm BPSi nanoparticles with different surface modifications by RAW 264.7 macrophages. Surface coatings are described in the Materials and Methods section. Left column: fluorescence from Cy5.5 conjugated to the particles. Middle column: cell membrane stained with CellMask. Right column: merged left and middle columns.

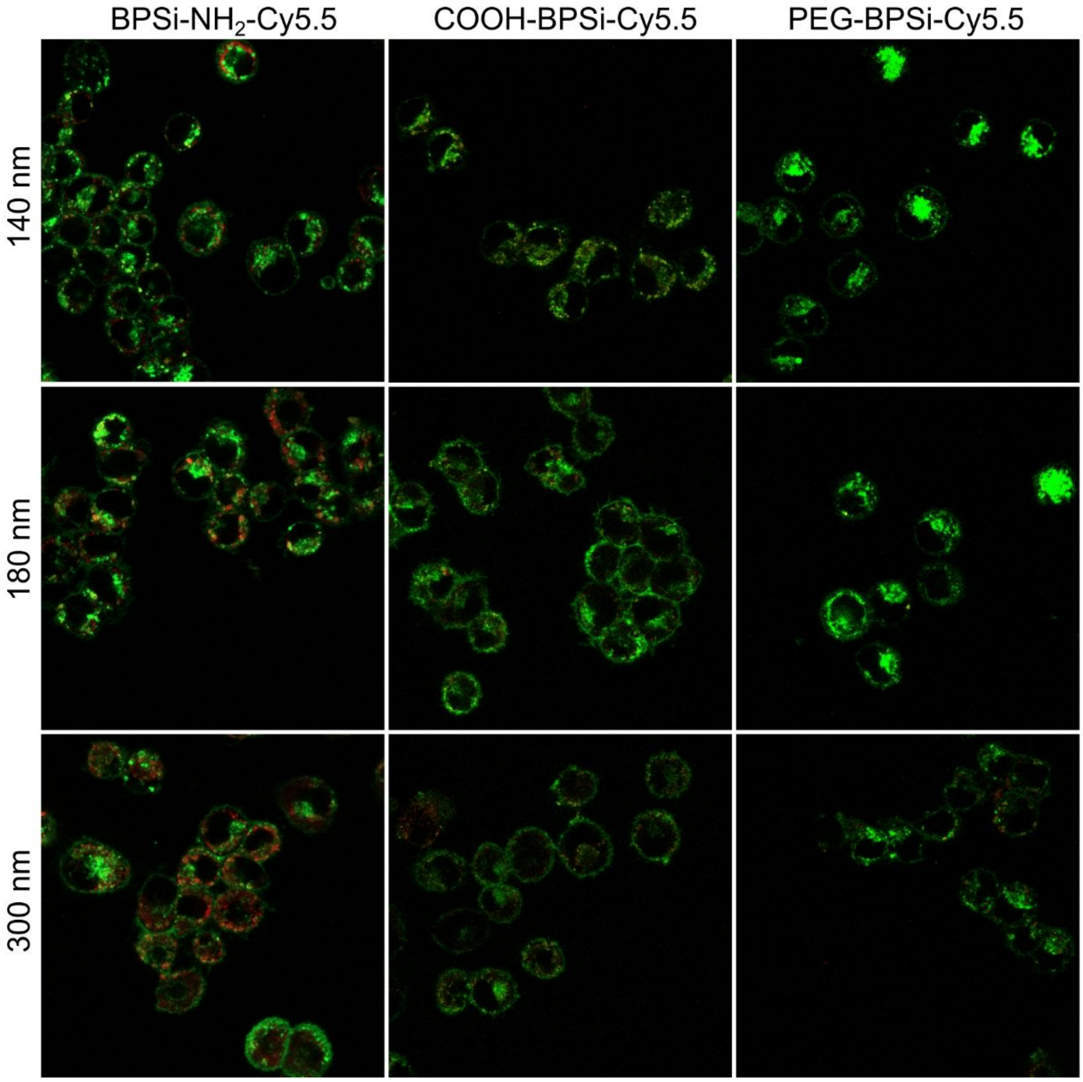

Figure S13. Comparison of internalization by RAW 264.7 macrophage cells of 140. 180 and 300 nm BPSi nanoparticles with different surface modifications. Surface coatings are described in the Materials and Methods section.

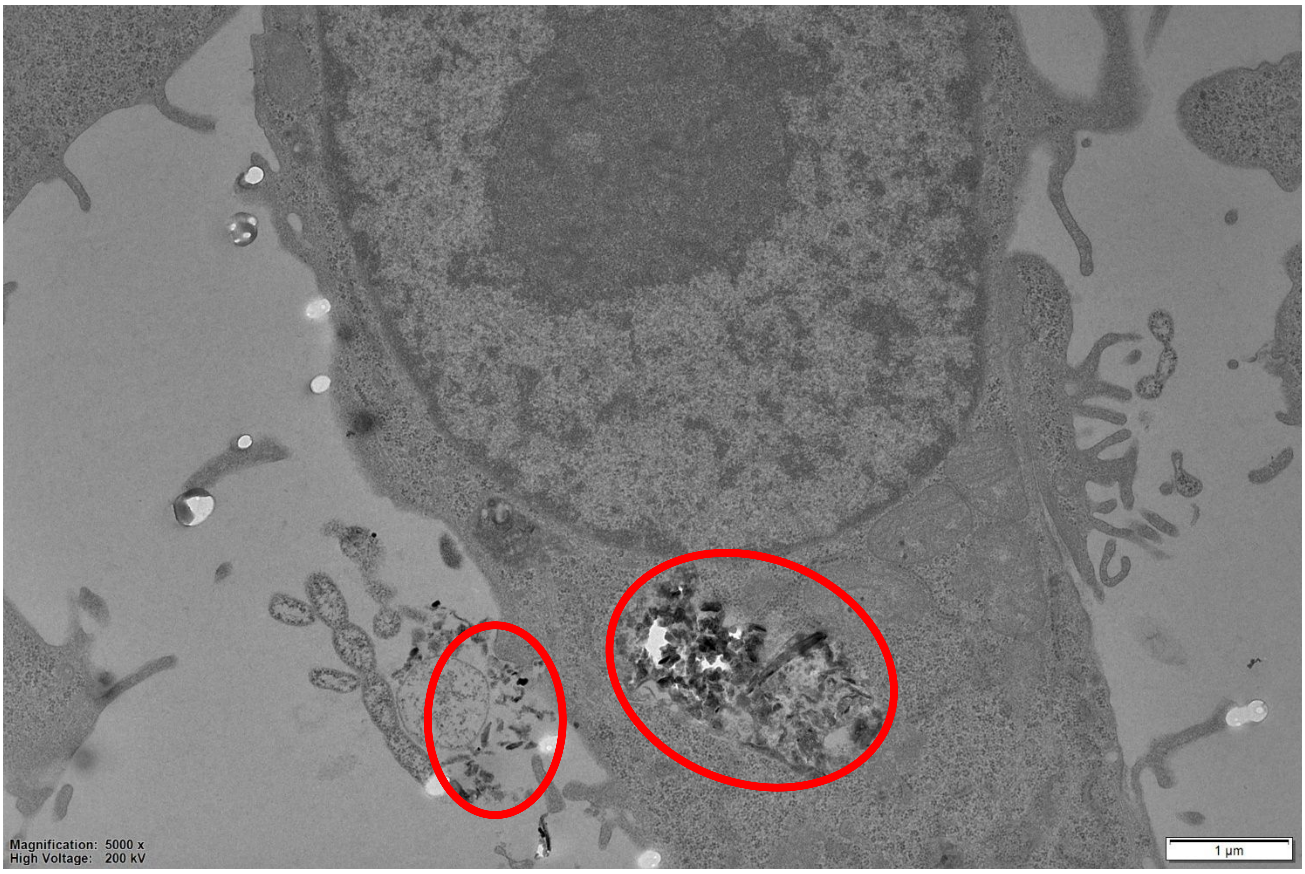

Figure S14. TEM images of 140 nm BPS-NH_2-Cy5.5 NPs after 24 h incubation with CT26 cells. The red circles show large patches of aggregated NPs both internalized into cells and aggregated near cell membrane.

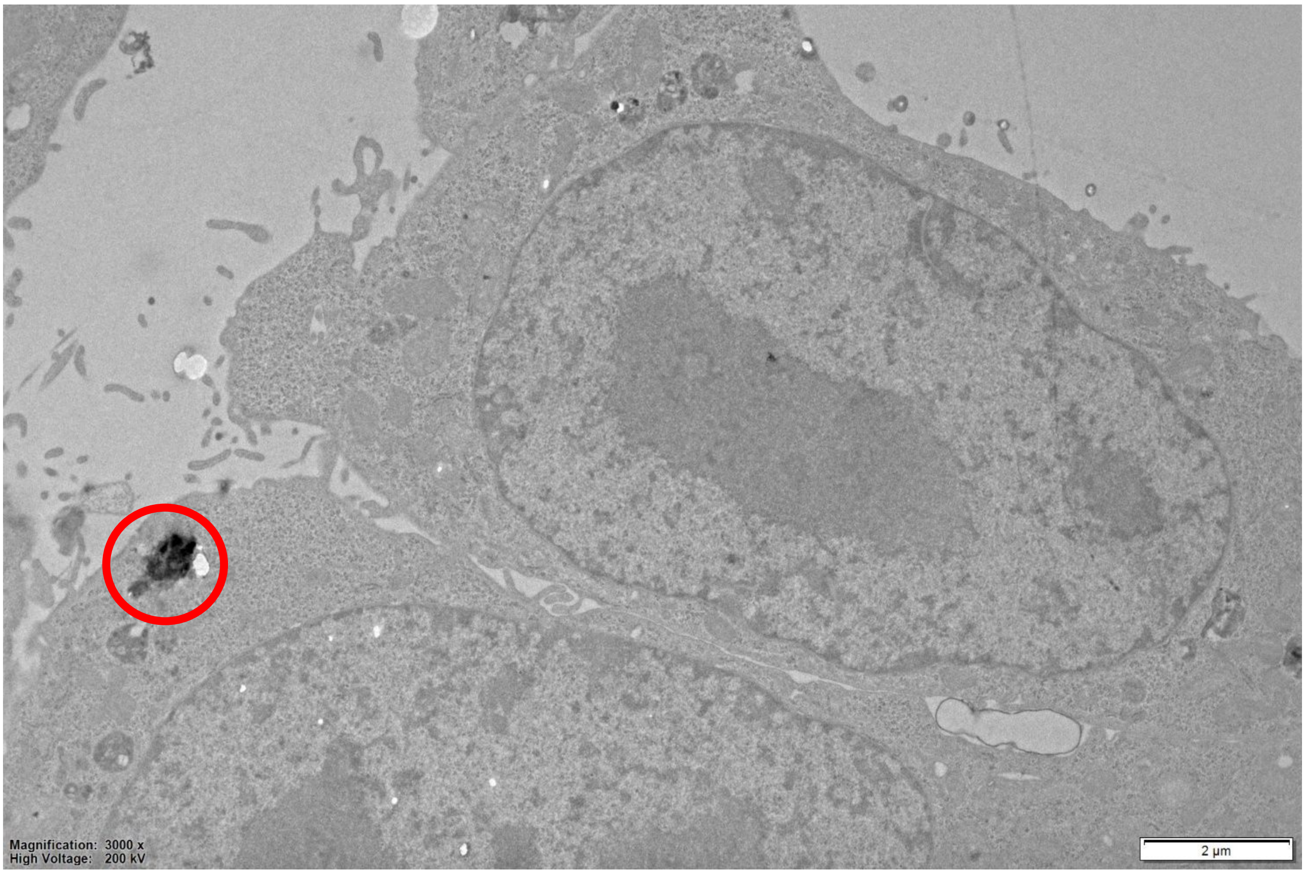

Figure S15. TEM images of 140 nm COOH-BPS-Cy5.5 NPs after 24 h incubation with CT26 cells. The red circles show large patches of aggregated NPs internalized into cells.

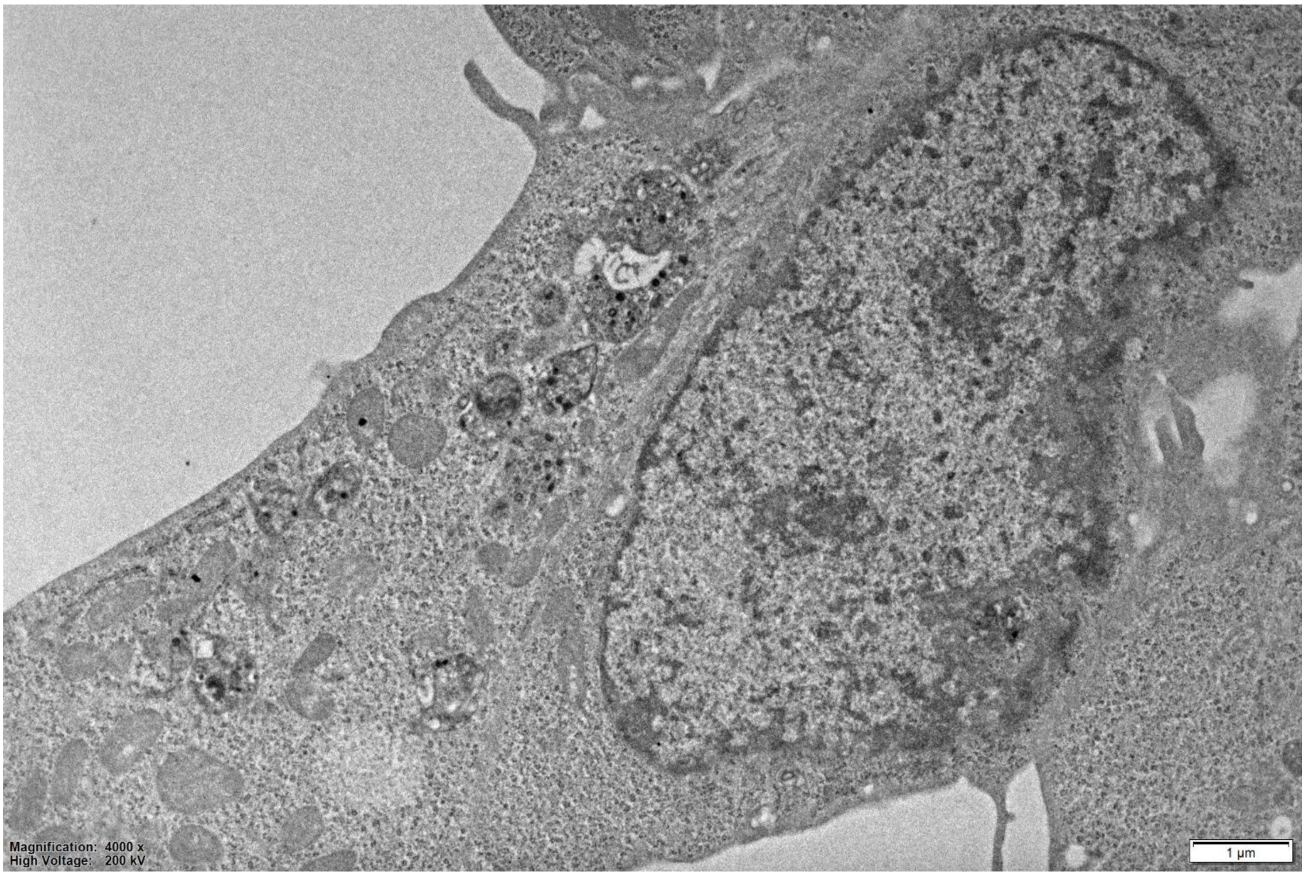

Figure S16. TEM images of 140 nm PEG-BPS-Cy5.5 NPs after 24 h incubation with CT26 cells.

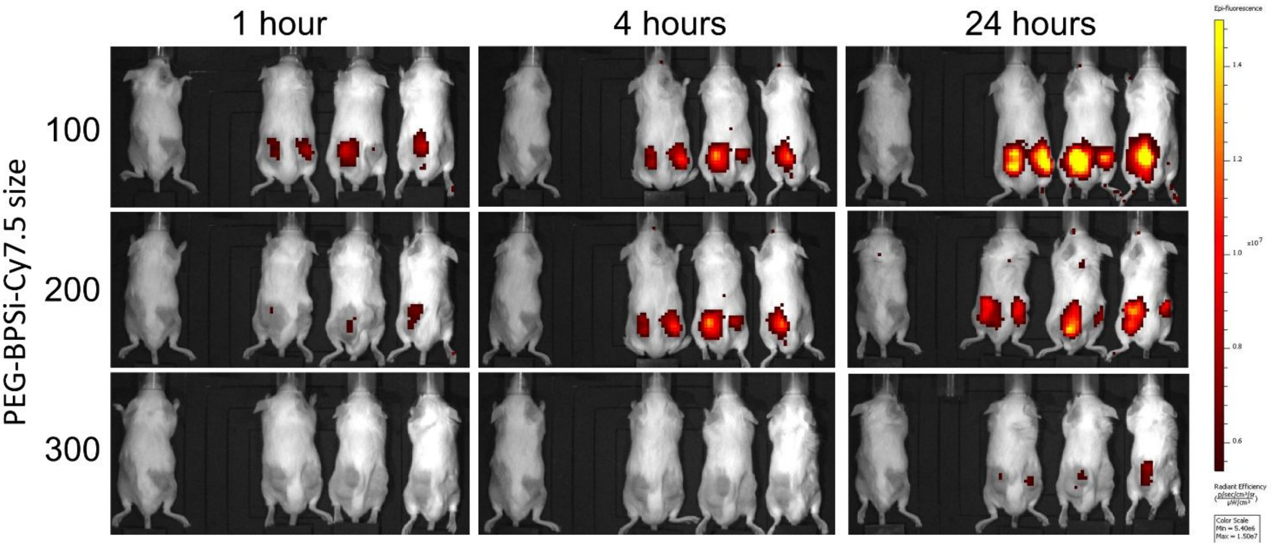

Figure S17. Distribution of 140 nm. 200 nm and 300 nm PEG-BPSi-Cy7.5 particles at different time points. Dorsal view; excitation: 740 nm; emission: 845 nm. Fluorescence signal comes from Cy7.5 dye conjugated to the particles during their accumulation in the tumors. Particles with smaller sizes demonstrate higher accumulation and higher fluorescence signal from tumors than larger particles.

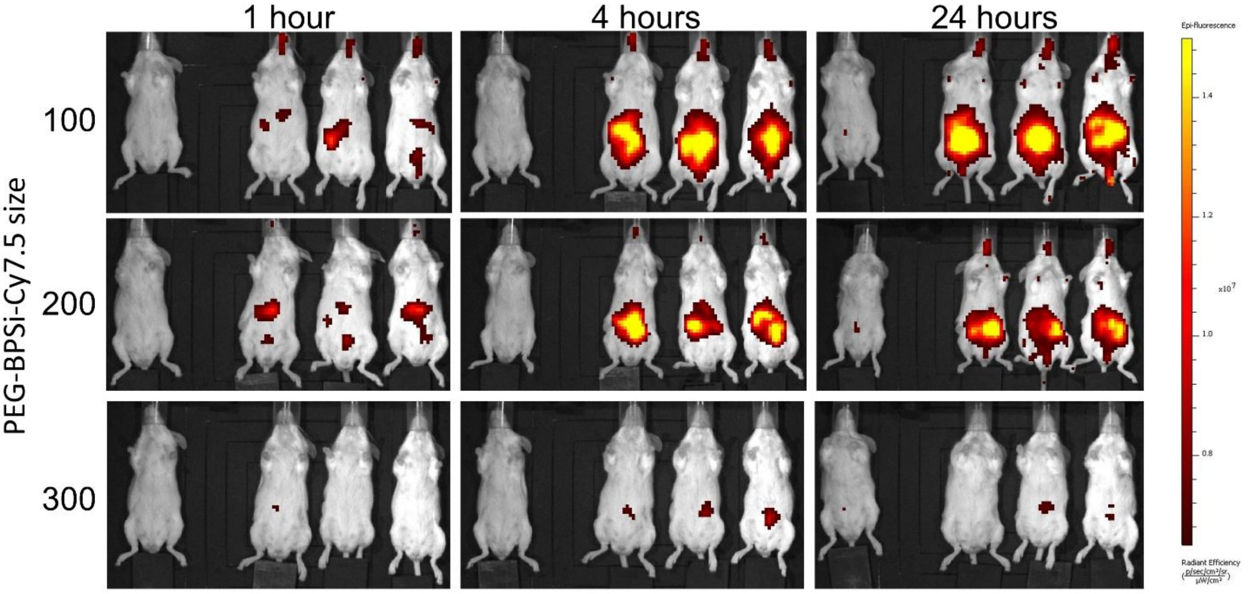

Figure S18. Distribution of 140 nm. 200 nm and 300 nm PEG-BPSi-Cy7.5 particles at different time points. Ventral view; excitation: 740 nm; emission: 845 nm. Most of the fluorescence signal comes from Cy7.5 dye conjugated to the particles during their accumulation in liver and spleen.

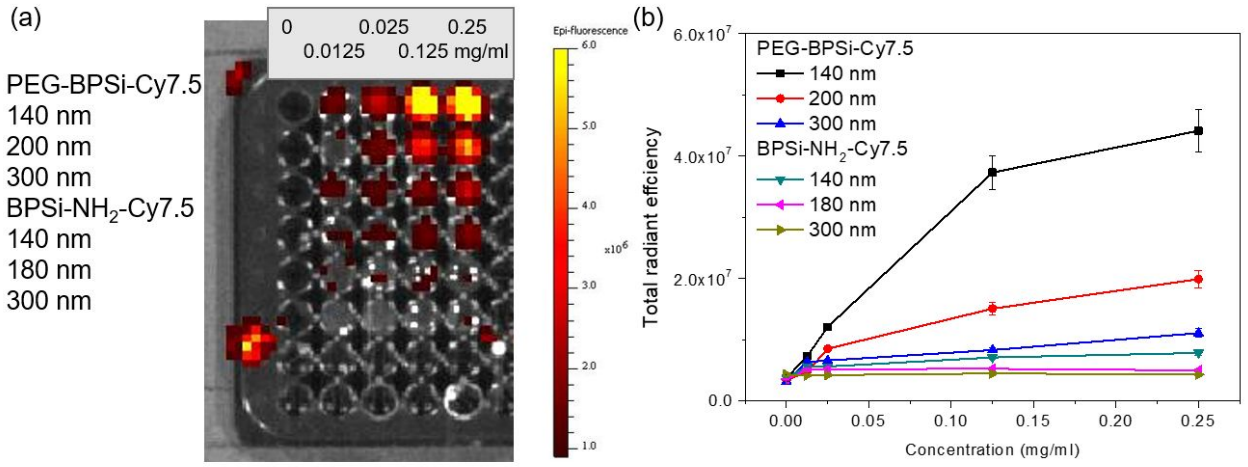

Figure S19. Evaluation of fluorescence from PEG-BPSi-Cy7.5 and BPSi-NH_2-Cy7.5 NPs of different sizes dispersed in PBS using IVIS. Excitation: 740 nm; emission: 845 nm. (a) Fluorescence from 200 µl of PEG-BPSi-Cy7.5 and BPSi-NH_2-Cy7.5 with different concentrations. (b) Total radiant efficiency calculated from each well after selecting appropriate ROIs in IVIS software.

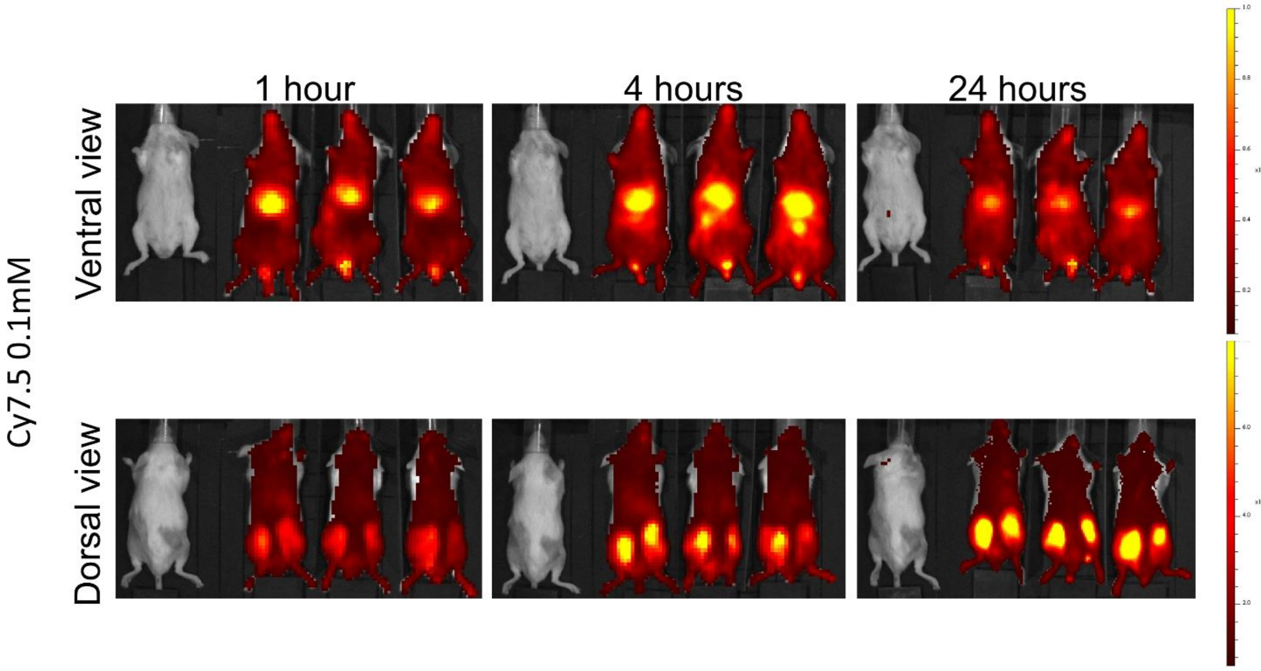

Figure S20. Fluorescence from Cy7.5 dye (0.1 mM in 200 µl of 5 % mannitol solution) at different time points. Ventral (upper row) and dorsal views (lower row). Excitation: 740 nm; emission: 845 nm.

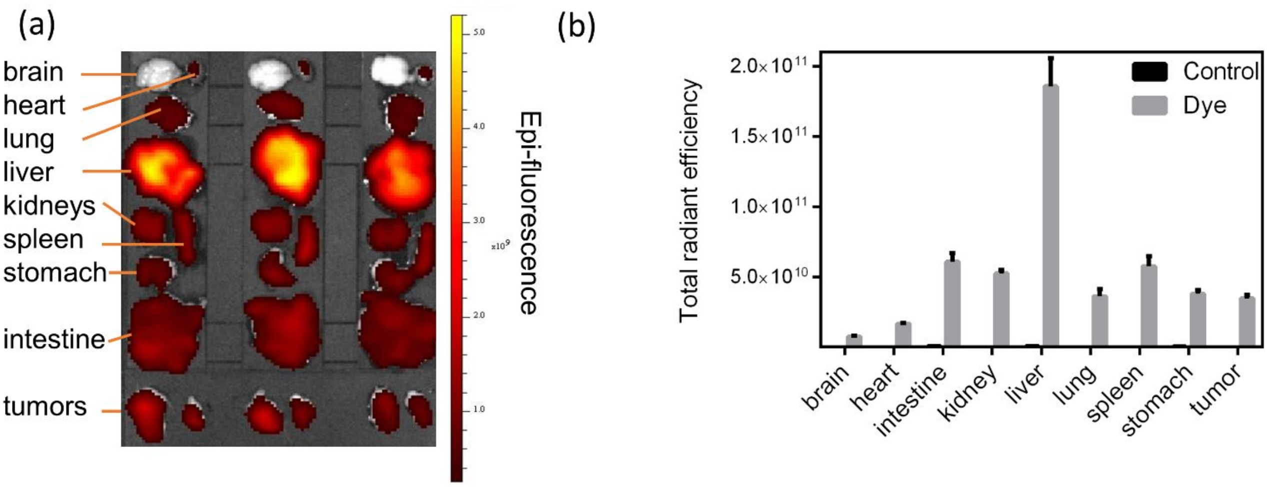

Figure S21. *Ex vivo* distribution of Cy7.5 (0.1 mM) dye 24 post i.v. injection. (a) Fluorescence from Cy7.5 dye. Excitation: 740 nm; emission: 845 nm. (b) Total radiant efficiency calculated using IVIS software after selecting ROIs corresponding to the organs.

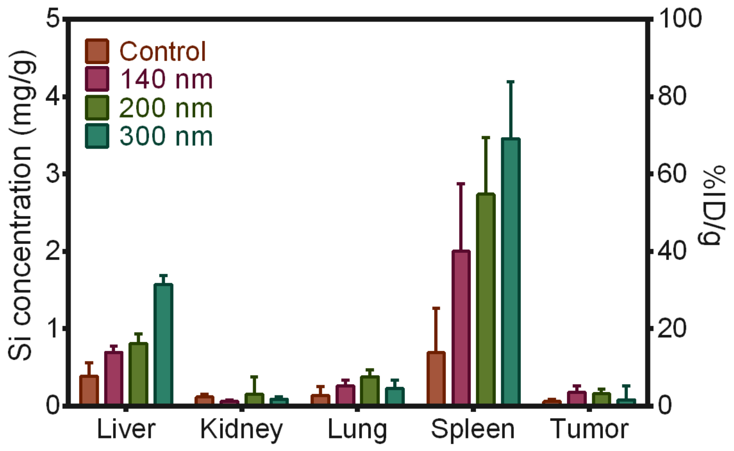

Figure S22. Si content in the major organs and tumors measured by ICP-OES. The data is presented as mass of Si in mg divided by the mass of each organ in g. N = 3.

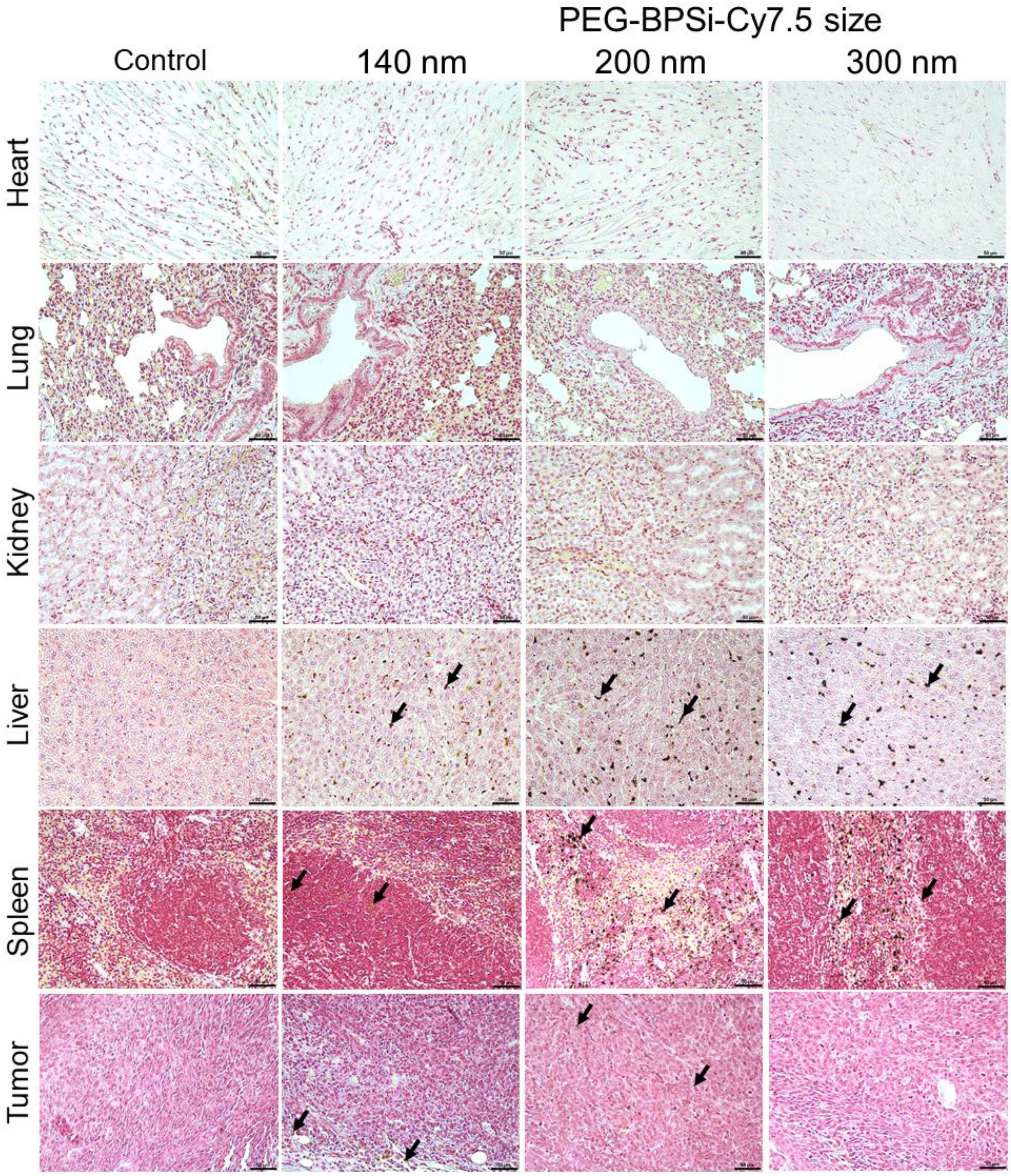

Figure S23. Histological examination of vital organs with neutral red staining. Arrows point to PEG-BPSi-Cy7.5 patches in spleen, liver, and tumor tissues. No large patches can be observed in lungs, kidneys, or heart. Scale bar is 50 µm.
